# Non-communicable diseases among adolescents: current status, determinants, interventions and policies

**DOI:** 10.1186/s12889-020-09988-5

**Published:** 2020-12-14

**Authors:** N. Akseer, S. Mehta, J. Wigle, R. Chera, Z. J. Brickman, S. Al-Gashm, B. Sorichetti, A. Vandermorris, D. B. Hipgrave, N. Schwalbe, Z. A. Bhutta

**Affiliations:** 1 Centre for Global Child Health, Hospital for Sick Children, Toronto, ON M5G 0A4 Canada; 2 Dalla Lana School of Public Health, University of Toronto, Toronto, Canada; 3 Division of Adolescent Medicine, Hospital for Sick Children, Toronto, Canada; 4 UNICEF, New York, USA; 5 Spark Consulting, New York, USA; 6 Center of Excellence in Women and Child Health, the Aga Khan University, Karachi, Pakistan

**Keywords:** Adolescents, Non-communicable diseases, Determinants, Policies, Risk factors

## Abstract

**Background:**

Addressing non-communicable disease (NCDs) is a global priority in the Sustainable Development Goals, especially for adolescents. However, existing literature on NCD burden, risk factors and determinants, and effective interventions and policies for targeting these diseases in adolescents, is limited. This study develops an evidence-based conceptual framework, and highlights pathways between risk factors and interventions to NCD development during adolescence (ages 10–19 years) and continuing into adulthood. Additionally, the epidemiologic profile of key NCD risk factors and outcomes among adolescents and preventative NCD policies/laws/legislations are examined, and a multivariable analysis is conducted to explore the determinants of NCDs among adolescents and adults.

**Methods:**

We reviewed literature to develop an adolescent-specific conceptual framework for NCDs. Global data repositories were searched from Jan-July 2018 for data on NCD-related risk factors, outcomes, and policy data for 194 countries from 1990 to 2016. Disability-Adjusted Life Years were used to assess disease burden. A hierarchical modeling approach and ordinary least squares regression was used to explore the basic and underlying causes of NCD burden.

**Results:**

Mental health disorders are the most common NCDs found in adolescents. Adverse behaviours and lifestyle factors, specifically smoking, alcohol and drug use, poor diet and metabolic syndrome, are key risk factors for NCD development in adolescence. Across countries, laws and policies for preventing NCD-related risk factors exist, however those targeting contraceptive use, drug harm reduction, mental health and nutrition are generally limited. Many effective interventions for NCD prevention exist but must be implemented at scale through multisectoral action utilizing diverse delivery mechanisms. Multivariable analyses showed that structural/macro, community and household factors have significant associations with NCD burden among adolescents and adults.

**Conclusions:**

Multi-sectoral efforts are needed to target NCD risk factors among adolescents to mitigate disease burden and adverse outcomes in adulthood. Findings could guide policy and programming to reduce NCD burden in the sustainable development era.

**Supplementary Information:**

The online version contains supplementary material available at 10.1186/s12889-020-09988-5.

## Background

The global burden of non-communicable diseases (NCDs) is a growing public health crisis that requires attention and action from the international community [1]. As the leading cause of mortality, this class of diseases is responsible for 38 million of 57 million annual deaths, with 85% of these deaths occurring in low- and middle-income countries (LMICs) [2]. However, the discussion on efforts to address NCDs to-date has focused on the adult population, with adolescents largely overlooked [3]. Since NCDs are often considered ‘lifestyle illnesses’ and the youth population is commonly thought of as healthy, modest efforts have been made to assess their health, disease prevention and lifestyle modification. Yet, adolescents experience a substantial share of the global NCD burden [4, 5].

Extensive research has shown that NCDs are primarily attributed to underlying and modifiable risk factors that often emerge during these earlier years [5–7]. It is estimated that approximately 70% of premature deaths occurring during adulthood are the result of health-related behaviours that are initiated in childhood and adolescence [3, 8]. Such risk factors, including overweight and obesity, physical inactivity, substance use and poor nutrition, substantially contribute to disease development and poor health in later life [8]. For example, the prevalence of overweight and obesity increases drastically during mid-adolescence and into adulthood [9]. Overweight and obesity during childhood and adolescence represents a significant risk for premature mortality and physical morbidity later in life, including cardiovascular disease, asthma, and certain types of cancers [10]. Drug and substance use also represent a threat for multiple health outcomes, including poor mental health [11]. Since health behaviours and risk exposures that emerge during adolescence underpin health and well-being across the life-course and also affect pregnancy outcomes, investments must be made in the health of current and future generations. In fact, the 2016 Lancet Commission on Adolescent Health and Well-being recommended investment in dominant NCD-related health behaviours among adolescents as a means of preventing future disease development [12].

Addressing NCDs has emerged as a global priority in the Sustainable Development Goals (target 3.4) [13] and the focus of a third UN high-level meeting [14], and includes the establishment and promotion of cost-effective interventions to prevent and address NCDs [15]. However, existing literature provides patchy insight into the current state of NCD-related lifestyle and behavioral risk factors among young people. In fact, to-date, there is no systematic assessment of the NCD burden, risk factors and determinants of NCDs, and effective interventions and policies for targeting these diseases in this population. Further, recent and ongoing efforts by the World Health Organization have involved the establishment of several global strategies, policies, laws and legislations to reduce the harmful use of alcohol [16], increase monitoring and decrease tobacco use [17], as well as to address childhood obesity [18]. However, limited evidence is available on the monitoring and evaluation, and effective implementation of these NCD-related efforts.

We conducted a comprehensive assessment of NCDs among adolescents, with the specific objective to: 1) develop an evidence-based conceptual framework explaining the determinants, pathways and interventions for NCDs among this age group and in later life; 2) describe the age- and sex-specific burden of major NCDs and risk factors among adolescents, globally and by geographical region; 3) examine major risk factors for adolescent NCDs and their role in adulthood NCD burden; 4) synthesize and summarize available evidence on effective interventions and delivery platforms to reduce the burden of NCDs among adolescents; 5) explore the availability of related laws and policies to reduce NCDs among adolescents globally and by geographical region; and 6) conduct multivariable assessment of key distal and intermediary contextual determinants of NCD morbidity and mortality among adolescents and, separately, the effect of these factors on adulthood NCD burden.

## Methods

We used a life-course approach and socio-ecological model to inform the development of a conceptual framework illustrating factors affecting NCDs in adolescence and in later life. We explored online databases for information pertaining to the burden of NCDs among adolescents, their determinants and risk factors, and relevant policies/interventions for NCDs in this population. Details are included in the Technical Additional file 1.

The proposed conceptual framework integrated several existing models and frameworks, including the WHO global monitoring framework [19], NCD framework for action/monitoring [20], socio-ecological models of adolescent health and development [2, 21] and key frameworks and determinants of adolescent health outlined in the 2012 and 2016 *Lancet* series on adolescent health [3, 20, 22]. The layout of the conceptual framework was adapted from the model on the life course approach to NCD prevention by WHO [20] and the multi-sectoral nutrition conceptual framework [23, 24]. An iterative process was conducted to identify, integrate and synthesize the concepts, development and structure of the conceptual framework.

Similar approaches were used to collect information on effective NCD-related interventions and laws/legislations/policies available for the prevention of these diseases during adolescence and in later life. The WHO Maternal, Newborn, Child and Adolescent Health (MNCAH) policy indicator database [25] contains data on eleven adolescent NCD-related risk and health outcome policies, laws and regulations from 104 low and middle income countries (LMICs) globally. We obtained and analyzed this data; details in the Technical Additional file 1.

We obtained national estimates on health and contextual indicators related to NCD development in adolescents and adults through a review of global data repositories performed from Jan 1st 2018 to July 6, 2018. We focused on 194 countries and assembled panel datasets from 1990 to 2015; we focused on this period for quantitative analysis to evaluate change and distribution across the Millennium Development Goal (MDG) period. Key areas of interest included distal factors (conflict, governance, population density, environment, urbanization, national wealth, health spending, telecommunications, infrastructure), factors intermediary to NCD outcomes (income inequality, women’s empowerment, health care services, youth empowerment, socioeconomic status), and proximal factors (i.e. behavioural, biological, nutritional and environmental risk factors). Primary data sources included the Global Health Observatory Data Repository [26], the State of the World’s Children global statistics database [27], the World Bank database [28], the United Nations Statistics Division [29] and the 2015 Global Burden of Disease (GBD) study housed at the Institute for Health Metrics and Evaluation (IHME) [30]. Estimates were obtained directly from the respective sources for each indicator without any manipulation. A summary of the hierarchical levels, domains and indicators with sources linked to NCD outcomes among adolescents and later adulthood in this study are included in the appendix (Additional file 1).

The major non-communicable diseases among adolescents were identified using disability-adjusted life years (DALY) data from the GBD. We constructed ranked estimates using national cause-specific DALYs across the six WHO regions (i. African region; ii. region of the Americas; iii. Eastern Mediterranean region; iv. European region; v. South East Asia region, and vi. Western Pacific region), two adolescent age categories (10–14 years, 15–19 years of age) and by sex. We also tabulated the NCD DALYS in late adolescence to adulthood (age 15–49 years, 50–69 years) that are attributable to lifestyle and behavioral risk factors that begin in the critical adolescence period.

We conducted two sets of multivariable analyses to understand the major contextual, social, economic and environmental determinants of NCD-related mortality and morbidity among 1) adolescents and 2) adults. This country-level ecological analysis used data for 195 countries obtained from the GBD data repository for the year 2015. For the adolescent model, the primary outcome was the country’s NCD DALY rates among 10–19 year old regressed onto a series of fixed-effect covariates. For the adult model, the outcome was country-level NCD DALY rates among 25–59 year olds which was regressed onto a series of contextual, social, economic and environmental factors. We could not run regression models of panel data or change from 1990 to 2015 since many countries did not have reliable estimates for key covariate indicators in 1990. We used a hierarchical modeling approach [20] with structural, national, community, household and individual level covariate indicators mapped to the respective levels described above (Additional file 1). We selected largely distal and intermediary factors that could have a differential impact on proximal risk factors of NCD development in adolescence and in later adulthood. Ordinary least squares regression models were fitted, and crude and standardized beta coefficients were estimated. Variables with skewed distributions were transformed appropriately to stabilize variance for regression analysis. Variables associated with the outcome at *p* < 0.20 in bivariate analysis were entered into multivariable model selection algorithms. Elastic net regression was used to select statistically significant variables (*p* < 0.15) in multivariable analysis at each level due to the large number of correlated predictors. Collinearity was assessed using variance inflation factors, and residual plots, influence/deviance statistics, AIC/BIC and adjusted R2 were consulted to assess model fit. Type 1 error rate was retained at 0.05 and analyses were conducted using SAS version 9.4 and RStudio Version 1.1.423.

## Results

### Conceptual framework

**Fig. 1:**
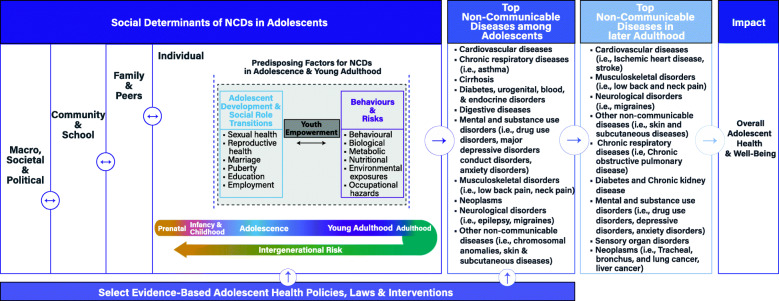
Conceptual framework on NCDs among adolescents

Our novel evidence-based conceptual framework (Fig. 1) represents a comprehensive and integrated approach to understanding the complex pathways through which risk and protective factors contribute to NCDs among adolescents and in later life. The framework provides a depiction of four key components: i) societal determinants; ii) individual predisposing factors across the adolescent and young adulthood stages of the life course; iii) top NCD conditions among adolescents and in later adulthood; and iv) select evidence-based policies, laws and interventions for this population. Key definitions, pathways and relationships of the NCD conceptual framework are detailed in Table 1 [31–35, 37].

**Table 1:** Conceptual framework for NCDs among adolescents

The evidence-based conceptual framework (Fig. 1) represents a comprehensive, integrated and novel approach to understanding the complex pathways through which structural, community, school, family, peer, and individual risk and protective factors contribute to the global burden of NCDs among adolescents and in later adulthood. *Key definitions* *Social Determinants of Health:* Conditions in which people are born, grow, develop, live, work and age. This includes the social, political, economic, environmental and cultural systems and forces that influence and shape the health and wellbeing of individuals. *Macro, Societal & Political*: Fundamental global and national social, economic, and political structures that shape inequalities in society, health and individual development. Macro trends include factors such as migration, conflict, environmental conditions, global economic development, technology and innovation. Societal and political determinants also represent underlying factors that substantially impact health, including national wealth, policies and laws, infrastructure, population density, governance, and culture. *Community & School*: Circumstances of daily life where adolescents live, work and learn, including youth and women’s empowerment, quality and access to health services, education, transportation, access to health services and employment opportunities and conditions. *Family & Peer Factors*: The primary protective and enabling structures that shape the health and development of young people. Relationships, connectedness, and the behaviours of family (e.g. parents, guardians, caretakers and siblings) and peers significantly influence the behaviour and health of adolescents. *Individual Factors:* Individual developmental milestones and social role transitions, including sexual and reproductive health, early marriage, age of puberty, education and employment. Behavioural, biological, metabolic, nutritional, environmental and occupational factors that are protective or risk factors for individual adolescent health. Macro, societal and political factors, including climate change and natural disasters, conflict, national wealth and health spending, infrastructure and urbanization, and governance represent critical overarching influences that shape the development and health of adolescents globally. These factors underlie and influence community and school factors, for example urbanization can improve young people’s access to education and health services, however may also increase young people’s risk for NCD-related risk factors, including mental health issues and obesity and physical inactivity [12]. Community and school level determinants play a substantial role in determining the current and future health of adolescents. Income inequality is associated with overall health outcomes, including mortality rates [9, 13] Inequalities in socioeconomic status also represent risks for NCDs including, increased physical inactivity, high BMI, poor psychological and physical well-being, high rates of substance use, bullying, and other poor behavioural and health outcomes [31, 32]. Access to education and educational attainment represent critical protective factors for health across the life-course. Better-educated individuals live longer and healthier lives globally, with lower mortality, and improved self-reported health outcomes [22, 33]. Diverse adolescent health outcomes and behaviours, including sexual and reproductive health, child marriage, mental health and self-harm [34] and obesity [33], are all positively influenced by increased educational attainment, particularly completion of secondary education. In addition, education significantly shapes the health of future generations, empowers youth and women, and narrows inequalities in status and health [35]. Availability and accessibility of health services represents a key approach to addressing and managing chronic health conditions and NCDs [36]. Youth unemployment and low-quality, unsafe, employment opportunities have been identified to significantly impact adolescents’ well-being, job satisfaction and health [37], including association with poor mental health, suicide and violence [22]. Family and peer connectedness, modeling of behaviours, and relationships represent significant protective or risk factors for adolescent health behaviours and outcomes, including smoking, violence, suicidal thoughts and behaviours, sexual and reproductive health, and overall healthy development [2, 36]. Connectedness and attitudes towards school have been associated with substance use, including drug, alcohol and tobacco [36]. Behaviours and risks vary across the life-course, and impact children and adolescents’ growth, development and risk for NCDs. Adolescence represents a time of significant biological, developmental and social role changes and transitions, including puberty, sexual and reproductive health, education, marriage, and employment. Furthermore, the initiation of behaviours such as tobacco use, poor diet, physical inactivity, and consumption of alcohol during childhood and adolescence contributes to the burden of disease during this time period, and substantially increases the risks for NCDs in later life. Mitigating and protective factors during adolescence include female empowerment and the empowerment of young people [35, 36]. The improved status of women in society (e.g. education, employment, increased age of marriage, etc.), has been associated with improved health outcomes for children and adolescents, while increased empowerment, education and employment of young people, are related to improved mental and physical health outcomes [22, 33, 34, 36]. The risk of developing NCDs increases across the life-course from childhood into adulthood [5], including increased risk of asthma, cardiovascular disease, diabetes, mental and substance use disorders (e.g. drug use disorders, major depressive disorder, etc.), musculoskeletal disorders, cancer, neurological disorders and other NCDs. Peer, family, community, national and broader global social determinants represent complex and interrelated factors that influence and shape individual behaviours and risks contributing to increased burden of NCDs. A supportive and enabling environment to develop and implement policies and interventions targeting structural, community, school, individual and crosscutting levels, represent a critical approach to improving adolescents’ health and development, and to addressing health behaviours and causes of NCDs (Appendix 7).	

### Burden and trends of NCD outcomes in adolescents

We explored the current burden of NCDs among adolescents by age and sex (Table 2), as well as by WHO region (Additional file 1). A large share of the burden of NCDs among 10–19 year olds is due to mental illnesses. Conduct disorder accounts for the most NCD DALYs (2595,245) among adolescents aged 10–14, representing 8% of the total NCD burden in this age group. In addition, anxiety disorders (2,100,974; 6%) and major depressive disorder (1,472,928; 4%) were the 3rd and 6th highest ranked NCD cause of DALYs among 10–14 year-olds, respectively. In 15–19 year olds, major depressive disorder is the top NCD cause of DALYs (3646,293), representing 8% of the total NCD burden in this age group. Comparable to the 10–14-year-old age group, anxiety disorders (2,510,537; 6%) and conduct disorder (1874,317; 4%) ranked 3rd and 7th, respectively, in the rank of top 10 NCDs with the highest DALY burden (Table 2).

**Table 2:** Distribution of NCD DALYs among adolescents by age and sex in 2015

		Both			Male			Female		
**Age group**	**Rank**	**Health Outcome**	**DALYs (1000s)**	**% of Total**	**Health Outcome**	**Total DALYs (1000s)**	**% of Total**	**Health Outcome**	**Total DALYs (1000s)**	**% of Total**
**10–14 years old**	**1**	Conduct disorder	2595	7.74	Conduct disorder	1638	9.42	Migraine	1273	7.89
	**2**	Asthma	2321	6.92	Asthma	1204	6.92	Anxiety disorders	1262	7.82
	**3**	Anxiety disorders	2101	6.26	Anxiety disorders	839	4.82	Asthma	1117	6.92
	**4**	Migraine	2046	6.10	Migraine	773	4.44	Conduct disorder	957	5.93
	**5**	Acne vulgaris	1476	4.40	Acne vulgaris	722	4.15	Major depressive disorder	825	5.11
	**6**	Major depressive disorder	1473	4.39	Low back pain	649	3.73	Acne vulgaris	753	4.67
	**7**	Low back pain	1270	3.79	Major depressive disorder	648	3.72	Low back pain	621	3.85
	**8**	Age-related and other hearing loss	1123	3.35	Age-related and other hearing loss	626	3.60	Age-related and other hearing loss	497	3.08
	**9**	Epilepsy	992	2.96	Epilepsy	539	3.10	Dermatitis	482	2.98
	**10**	Dermatitis	906	2.70	Autism	454	2.61	Epilepsy	453	2.81
		**Percent of total NCD burden in age and sex**		**48.60**	**Percent of total NCD burden in age and sex**		**46.51**	**Percent of total NCD burden in age and sex**		**51.04**
		NCD DALYs	33,546		NCD DALYs	17,398		NCD DALYs	16,147	
		Total DALYs	70,005		Total DALYs	38,135		Total DALYs	31,870	
**Age group**	**Rank**	**Health Outcome**	**DALYs (1000s)**	**% of Total**	**Health Outcome**	**Total DALYs (1000s)**	**% of Total**	**Health Outcome**	**Total DALYs (1000s)**	**% of Total**
**15–19 Years Old**	**1**	Major depressive disorder	3646	8.16	Major depressive disorder	1589	6.98	Major depressive disorder	2058	9.38
	**2**	Migraine	2709	6.06	Low back pain	1323	5.81	Migraine	1673	7.63
	**3**	Anxiety disorders	2511	5.62	Conduct disorder	1232	5.41	Anxiety disorders	1518	6.92
	**4**	Low back pain	2478	5.54	Acne vulgaris	1065	4.68	Low back pain	1155	5.27
	**5**	Acne vulgaris	2151	4.81	Migraine	1036	4.55	Acne vulgaris	1086	4.95
	**6**	Other musculoskeletal disorders	1923	4.30	Anxiety disorders	993	4.36	Other musculoskeletal disorders	1051	4.79
	**7**	Conduct disorder	1874	4.19	Asthma	889	3.90	Asthma	865	3.95
	**8**	Asthma	1755	3.92	Other musculoskeletal disorders	872	3.83	Conduct disorder	643	2.93
	**9**	Epilepsy	1150	2.57	Epilepsy	682	3.00	Age-related and other hearing loss	486	2.21
	**10**	Age-related and other hearing loss	1123	2.51	Age-related and other hearing loss	638	2.80	Epilepsy	468	2.13
		**Percent of total NCD burden in age and sex**		**47.69**	**Percent of total NCD burden in age and sex**		**45.32**	**Percent of total NCD burden in age and sex**		**50.16**
		NCD DALYs	44,706		NCD DALYs	22,771		NCD DALYs	21,935	
		Total DALYs	85,151		Total DALYs	46,335		Total DALYs	38,816	

DALYs due to NCD causes also vary by sex (Table 2). In 10–14 year olds, conduct disorder contributes the most NCD DALYs for males (1638,150; 9%), followed by asthma (1204,058), anxiety disorders (838,606), migraine (773,026) and acne vulgaris (722,333). In females of the same age, migraine contributes the greatest burden of disease (1273,422; 8%), followed by anxiety disorders (1262,368), asthma (1117,023), conduct disorder (957,096) and major depressive disorder (825,013). In 15–19 year olds, major depressive disorder contributes the most NCD DALYs for both males (1,588,775) and females (2,057,518); however, it does represent a greater proportion of all DALYs in females (9%) than in males (7%) in this age group. Overall, in both adolescent age groups, conduct disorder plays a larger role in males than females, and anxiety disorders and major depressive disorders play a larger role in females.

Across global regions, conduct disorder persists as the leading NCD among males 10–14 years of age (Additional file 1). In females of the same age, migraines are the most prominent NCD in the Eastern Mediterranean and South East Asia regions, anxiety disorders are the most prominent in the Americas and European and Western Pacific regions, and asthma is the most prominent NCD among girls in Africa. Among males 15–19 years of age, major depressive disorder is the leading NCD in Africa, the Americas, the Eastern Mediterranean, and South East Asia, whereas low back pain and acne vulgaris are the leading NCDs in Europe and in the Western Pacific respectively. Similarly, among females 15–19 years of age, depression contributes the most NCD DALYs in four of the six regions (Africa, Europe, Americas, Western Pacific), whereas migraine leads in the Eastern Mediterranean and South East Asia regions (Additional file 1).

### Burden, Behavioural, and socioeconomic risk factors

Select behavioural, lifestyle and socioeconomic risk factors for NCDs from adolescence into adulthood in regions around the world are highlighted in Table 3. With regards to lifestyle and behavioural factors, the prevalence of alcohol drinking among 15–19 year olds is more common among boys than girls, and is highest in regions of the Americas and Europe (> 50%). Similarly, tobacco use among young adolescents is higher among males compared to females, and ranges from 12 to 2% across regions. Insufficient physical activity is pervasive in almost 80% or more of adolescents aged 11–17 years across all regions and both sexes. The prevalence of overweight children and adolescents under 20 years of age is higher among males compared to females across all regions, the region with largest rate for both genders was the Americas with more than a third of the under 20 population being classified as overweight. Obesity in children and adolescents under 20 years of age is also higher among males and is highest in the regions of Middle East and North Africa and Europe. Youth literacy rates are high (almost 90% or more) in most regions but lag behind in Africa and South Asia. Secondary school enrollment rates are also consistent across most regions (70% or more) except for Eastern and Southern Africa and South Asia regions. Youth literacy and secondary school enrollment rates are similar between genders and this similarity is synonymous across all regions. Unemployment among youth 15–24 years is highest (28%+) in the MENA region followed by the Americas (18.5%) and Europe regions (18.5%).Breastfeeding patterns have been linked to overweight, type 2 diabetes, and possibly high blood pressure and cholesterol in childhood and adolescence, and even into adulthood [39, 40]. About 50% of South Asia and Eastern and Southern Africa exclusively breastfeeds child younger than 6 months, while only 28 to 39% of those in other regions practice exclusive breastfeeding. Sanitation is part of the broad set of environmental factors that directly and indirectly influence risk of NCDs in childhood and adolescence. Poor sanitation can result in, for instance, diarrhea, which can impact nutrient absorption and disease burden. It’s also linked to individual and social perceptions about health and wellbeing, which can influence an adolescent’ mental health [36, 41]. Access to improved water (53–62%) and improved sanitation (27–30%) facilities is notably lower in Africa while all other regions have > 80% availability.

**Table 3:** Distribution of select NCD risk factors among adolescents by global region

Risk Factor Indicators		Sex	Regions^1^							Data Source	Year of Data Collection
			Americas	Europe	Eastern and Southern Africa	Western and Central Africa	Middle East & North Africa	Pacific	South Asia		
**Behavioural Risk Factors**	Prevalence of current adolescent drinkers aged 15–19 (%) [26]	Males	54.9	69	38.5	34.5	14.1	25.2	9	WHO	2010
		Females	37.7	48.7	25.9	22.2	11.1	15.3	4.3		
	Prevalence of current smokers of cigarettes aged 13–15 per 100 population (%) [26]	Males	17.4	14.5	7.4	7.3	10.2	9.9	5.1	WHO	2008–2010
		Females	19.1	9.9	3.2	2	2.4	1.9	2		
	Prevalence of current tobacco use among adolescents aged 13–15 years (%) [26]	Males	17	–	–	–	21.3	12.4	21	WHO	2007–2014
		Females	13.8	–	–	–	9.7	3.5	7.4		
	Prevalence of insufficient physical activity (school-going adolescents 11–17 years) [26]	Both Sexes	81.2	83.2	85.2*		87.5	85	73.4	WHO	2010
		Males	87.1	87.7	87.9*		91	88.9	74.6		
		Females	75.3	78.4	82.3*		84.7	81	72.5		
**Biological Risk Factors**	Low birthweight (%) [27]	Both Sexes	9	6	–	–	–	–	–	UNICEF	2011–2016
**Nutrition Risk Factors**	Early initiation of breastfeeding (%) [27]	Both Sexes	54	57	63	40	40	43	39	UNICEF	2011–2016
	Introduction to solid, semi-solid or soft foods 6–8 months (%) [27]	Both Sexes	82	69	75	68	63	69	56	UNICEF	2011–2016
	Exclusive breastfeeding (< 6 months, %) [27]	Both Sexes	38	30	55	29	32	28	52	UNICEF	2011–2016
	Underweight, moderate and severe, under-5 (%) [26]	Both Sexes	1.6	–	17.2*		12.8	2.7	26.2	WHO	2017
	Stunting, moderate and severe, under-5 (%) [27]	Both Sexes	11	6	34	34	15	9	36	UNICEF	2011–2016
	Wasting, moderate and severe, under-5 (%) [27]	Both Sexes	1	2	7	9	7	3	16	UNICEF	2011–2016
	Overweight, moderate and severe, under-5 (%) [27]	Both Sexes	7	13	4	4	11	6	4	UNICEF	2011–2016
	Prevalence of overweight among children and adolescents, 5–19 years (%) [27]	Males	34.6	28.1	–	–	20.2	30.4	9.6	UNICEF	2016
		Females	32.6	24.2	–	–	20.7	18.8	8.1		
	Obese, under 20 years (%)	Males	5	7.4	3.9	4.4	20.7	3.8	2.5	Ng, 2014	2013
		Females	4.7	6.3	4	3.2	11	3.5	2.6		
**Socioeconomic Status**	Youth literacy rate, aged 15–24 years (%) [27]	Males	98	100	87	69	91	99	88	UNICEF	2011–2016
		Females	99	99	85	55	88	97	80		
	Primary school, net attendance ratio (%) [27]	Males	96	94	78	72	94	97		UNICEF	2008–2013
		Females	96	95	79	68	93	97			
	Secondary school, net enrolment ratio (%) [27]	Males	74	93	29	–	74	71	63	UNICEF	2011–2016
		Females	77	92	30	–	74	76	66		
	Out-of-school rate of children of primary school age (%) [27]	Males	5	4	17	–	6	6	5	UNICEF	2011–2016
		Females	4	4	19	–	8	6	6		
	Unemployment, youth total (% of total labor force ages 15–24) [28]	Both Sexes	18.5	18.5	14.2		28.1	10.3	10.4	World Bank	2017
	Child labour (%) [27]	Both Sexes	11	–	26	32	7	–	–	UNICEF	2010–2016
**Access to Improved Water and Sanitation**	Improved water, total (% of population with access) [27]	Both Sexes	96	95	53	62	93	94	88	UNICEF	2015
	Improved sanitation facilities, total (% of population with access) [27]	Both Sexes	86	93	30	27	89	77	46	UNICEF	2015
**Health Care Services and Essential Commodities/Medicine**	Skilled birth attendance, aged 15–49 years (%) [27]	Both Sexes	96	99	60	52	86	95	73	UNICEF	2013–2016
	Measles (MCV immunization on coverage among 1 year olds) (%) [27]	Both Sexes	92	93	76	67	89	93	84	UNICEF	2016
	DPT3 immunization coverage among 1-year olds (%) [27]	Both Sexes	90	90	80	67	88	94	86	UNICEF	2016
	Antenatal care coverage (4+ visits) (aged 15–49 years) (%) [27]	Females	90	87	52	52	66	74	46	UNICEF	2016
**Fertility Rates/Women Empowerment**	Adolescent birth rate, number of births per 1000 adolescent girls aged 15–19 years [27]	Females	74	29	113	130	41	21	44	UNICEF	2009–2014
	Percent of women giving birth by age 18 (%) [27]	Females	19	4	26	29	8	7	20	UNICEF	2011–2016
	Married or in-union women of reproductive age who have their need for family planning satisfied with modern methods (%) [26]	Females	83	75.1	52.2*		63.6	89.7	75.1	WHO	2018
	Unmet need for family planning (%) (aged 15–49 years) [26]	Females	9.4	10.4	24.4*		17.7	5.8	13.3	WHO	2010
**Gender Inequality**	Percent of women aged 20–24 years who were married by age 15 (%) [27]	Females		1	9	14	3	2		UNICEF	2010–2016
	Percent of women aged 20–24 years who were married by age 18 (%) [27]	Females		11	35	41	17	15		UNICEF	2010–2016
	Prevalence of FGM/C (% of girls and women of reproductive age 15–49 years experiencing FGM/C) [27]	Females			45	31				UNICEF	2004–2016
**Income Inequality**	GNI per capita (U.S.$) [38]		8200	22,651	1454**		7246	10,170	1743	World Bank	2017
**Urbanization**	% of total population urbanized [27]		80	64	31	45	63	57	33	UNICEF	2016

^1^ Regions are based on a combination of the seven UNICEF regions (Southern and Eastern Africa, West and Central Africa, Caribbean, Europe & CIS, North Africa, Pacific, South Asia and Southern Africa) and six WHO regions (Africa, Americas, Europe, Mediterranean, South-East Asia, and Western Pacific) and when necessary data from the World Bank regions (East Asia & Pacific, Europe & Central Asia, Latin America & Caribbean, Middle East & North Africa, Sub-Saharan Africa, South Asia). The amalgamation of regions includes: the Americas (Caribbean UNICEF region, Americas WHO region, Latin America and Caribbean World Bank region), Europe region (Europe & CIS UNICEF region, Europe WHO region and Europe & Central Asia World Bank region), Middle East and North Africa region (Middle East and North Africa UNICEF region, Eastern Mediterranean Region WHO and Middle East & North Africa World Bank region), Pacific region (Western Pacific WHO region, Pacific UNICEF region and East Asia & Pacific World Bank region), Southern and Eastern Africa (Southern and Eastern Africa region UNICEF), Western and Central Africa region (UNICEF) and South Asia region (South Asia UNICEF region, South East Asia WHO region and South Asia World Bank region)

* = Regional estimates are only available for the WHO African region

** = Regional estimates are only available for World Bank Sub Saharan Africa region

+ = Regional estimates were calculated by weighting country estimates with population data from the respective year (e.g. 2010) using the United Nations population prospects database and incorporating those into a combined estimate for the region

- = Regional data unavailable

Death and disability are associated with risk factors that begin in adolescence, although causality cannot be inferred from these data (Additional file 1). The top causes of premature death among 15–49 year olds are related to high body mass index (e.g. higher body adiposity), high blood pressure, high plasma blood glucose (diabetes), high cholesterol, smoking, drug and alcohol use, diets low in fruits and whole grains, and air pollution. These top factors were not different between sexes. These important risk factors even remained as the top contributors of later adulthood (50–69 years) disease-related disability and death (Additional file 1).

### Multivariable determinants of NCDs among adolescents and adults

In our analysis of correlates of NCD DALYS among adolescents, at the distal level, macro factors such as indicators of conflict and humanitarian crises (e.g. refugee populations, *p* < 0.0001), and state governance (effectiveness [*p* < 0.0001], corruption [*p* < 0.0001]) were independently associated with higher NCD burden (Table 4). Across a range of statistically significant national structural factors at the intermediate I level, urbanization (R2 = 14%, *p* < 0.0001), GDP per capita (R2 = 11%, *p* < 0.0001), and total health expenditure per capita (R2 = 12%, *p* < 0.0001) were more strongly related to the outcome. At the intermediate II level, other indicators of household socioeconomic status, youth literacy and fertility rates, proxies of women’s empowerment, income inequities, and availability of human resources for health were all statistically significant correlates of adolescent NCDs. In multivariable analyses, a final joint model explained about 62% of the variance in adolescent NCD DALYs worldwide. This included a series of distal and intermediate factors such as the total refugee populations in a country, effective state governance, urbanization, access to electricity, population density, total health expenditure per capita, adolescent fertility, and a proxy of female gender empowerment (secondary school gender parity index) (Table 4).

**Table 4:** Hierarchical bivariate and multivariable determinants of NCD DALYs among adolescents in 194 countries, 2015

Domain/Indicator		Outcome: DALYs attributed to NCDs among adolescents (rate, 2015)			
		Bivariate			Multivariable
		R-Square	Β estimate (Standard error) *P*-value	StB	Β estimate (Standard error) *P*-value
**Level 3 Model- Distal: Macro Structural Factors** ^**a**^					
**Conflict**	**Source & Year**				
Battle related deaths (total; log)	World Bank, 2013	1%	−23.5 (16.92) 0.1673	− 0.10	–
Internally displaced persons (total; log)	World Bank, 2013	1%	−21.8 (24.92) 0.3838	− 0.07	–
Refugee populations by country of asylum (total; log)	World Bank, 2014	16%	−58.3 (10.31) <.0001	− 0.39	−19.8 (8.13) 0.0156
**Governance**					
Political stability/absence of terrorism	World Bank, 2013	0%	−11.3 (60.98) 0.8534	−0.01	–
Government effectiveness ^1^	World Bank, 2013	11%	−264.4 (56.03) <.0001	−0.33	− 442.4 (44.27) <.0001
Corruption ^1^	World Bank, 2013	10%	−245.9 (56.74) <.0001	−0.31	–
**Environment**					
Frequency of natural disasters (total; log)		0%	−72.2 (84.02) 0.3914	−0.07	–
Cost damage of natural disasters (USD; log)		2%	−11.7 (6.91) 0.0924	−0.13	–
					**Distal Model R2:** **38%**
**Level 2 Model- Intermediate I: National Structural Factors** ^**b**^					
**Infrastructure and Urbanization**					
Urbanization (% of population; log)	UNICEF, 2016	14%	− 597.0 (111.74) <.0001	−0.37	− 261.0 (120.39) 0.0316
Access to electricity (% population; cubed)	World Bank	9%	−0.00057 (0.00014) <.0001	−0.30	− 0.0005 (0.00016) 0.0014
**Population Density**					
Population density (people per m^2^ land; log)	World Bank	2%	−91.2 (43.15) 0.036	−0.16	−52.8 (33.41) 0.1162
**Telecommunications Access**					
Mobile cellular subscriptions (per 100 people; log)	World Bank, 2014	3%	− 266.1 (114.17) 0.0209	−0.17	–
Internet users (per 100 people; log)	World Bank, 2014	6%	−147.7 (43.14) 0.0008	−0.25	–
**National Wealth**					
GDP per capita, 5 year lag (USD; log) ^3^	World Bank, 2014	11%	−172.9 (37.70) <.0001	−0.33	–
**Health Spending**					
Government expenditure on health, 2 year lag (% of total health expenditure; log)	WHO, 2013	1%	−165.0 (151.16) 0.2766	−0.08	–
Total health expenditure per capita, 2 year lag (PPP, NCU per USD; log) ^3^	WHO, 2013	12%	− 169.7 (34.83) <.0001	−0.34	−111.3 (62.56) 0.0771
					**Intermediate I Model R2:** **47%**
**Level 3- Intermediate II: Community, Household & Individual Factors** ^**c**^					
**Socioeconomic Status (Education and Employment)**					
Adult literacy rate (% of adults ages 15+ years; cubed)	World Bank	1%	−0.00024 (0.00022) 0.2767	−0.10	
Primary school enrolment ratio (gross %; log)	World Bank, 2003–2014	1%	719.0 (499.43) 0.1519	0.11	
Secondary school enrolment ratio (gross %; log)	World Bank, 2013	7%	− 496.9 (144.18) 0.0007	−0.27	
Employment to population ratio (% of adults 15+ years; log)	World Bank	4%	724.5 (282.59) 0.0112	0.19	
**Youth Empowerment**					
Youth literacy rate (% total 15–24 year olds; squared)	World Bank, 2014	3%	−0.1 (0.03) 0.0718	−0.16	–
Female youth literacy rate (% 15–24 year olds; squared)	World Bank, 2014	2%	−0.04 (0.03) 0.1169	− 0.14	–
Youth unemployment rate (% total 15–24 year olds; log)	World Bank, 2013	1%	−95.3 (76.09) 0.212	− 0.10	–
Adolescent fertility rate (births per 1000 females aged 15–19 years; log)	World Bank, 2009–2014	12%	256.0 (52.22) <.0001	0.34	224.4 (66.17) 0.0009
**Women Empowerment and Gender Equity**					
Total fertility rate (births per woman; log)	World Bank, 2013	7%	454.5 (122.04) 0.0003	0.27	–
Adult female literacy rate (% females 15+ years who can read and write; cubed)	World Bank	0.5%	−0.016 (0.021) 0.4481	−0.07	–
Women in parliament (% of parliamentary seats held by women; log)	World Bank, 2014	4%	−92.3 (35.97) 0.0111	−0.19	–
Secondary school gender parity index (ratio of girls to boys in secondary education; log)	World Bank, 2013	3%	− 877.5 (416.20) 0.0366	−0.17	− 700.4 (341.58) 0.0422
Tertiary school gender parity index (ratio of girls to boys in tertiary education; log)	World Bank, 2013	6%	− 206.4 (66.82) 0.0024	− 0.25	–
**Income Equity**					
GINI index (log)^**2**^	World Bank, 2012	12%	1072.6 (269.17) 0.0001	0.35	–
**Access to Health Services and Commodities**					
Out of pocket expenditure as % of total health expenditure (log)	WHO, 2013	0.06%	−23.7 (75.81) 0.7544	−0.02	–
Physician density per 1000 population (log)	WHO, 2003–2013	10%	− 177.1 (47.11) 0.0003	− 0.32	–
					**Intermediate II Model R2:** **62%**

Note: variables significant at *p* < 0.20 in bivariate analysis were entered into elastic net linear regression models; ^1^ Government effectiveness and corruption were strongly collinear (> 80%) and thus only the former was entered into multivariable modeling; ^2^ Due to small sample size (*n* = 117 countries), GINI index not considered in multivariable analysis; ^1^ GDP per capita and health expenditure per capita were strongly collinear (> 80%) and thus only the latter was entered into multivariable modeling

^a^ Level 3 multivariable model includes all statistically significant (*p* < 0.15) distal variables as listed

^b^ Level 2 multivariable model includes level 3 model+ all statistically significant (*p* < 0.15) intermediate I variables as listed

^c^ Level 1 multivariable model includes level 2 model+ all statistically significant (*p* < 0.15) intermediate II variables as list

Our model of correlates of NCD DALYs among adult populations is displayed in Table 5. Distal level macro factors associated with the burden of NCDs among adults in bivariate analyses were government effectiveness [*p* = 0.0003] and corruption [*p* = 0.0119]). At the intermediate I level, access to electricity (R2 = 5%, *p* < 0.0001), mobile cellular subscriptions (R2 = 15%, *p* < 0.0001), GDP per capita (R2 = 9%, *p* < 0.0001), and total health expenditure per capita (R2 = 8%, *p* < 0.0001) were strongly related to NCD burden among adults. The final adjusted model for adult populations included the distal and intermediate factors of corruption in state governance, urbanization, access to electricity, GDP per capita, secondary enrolment ratio, physician density per 1000 population as well as proxies for female gender empowerment (secondary and tertiary school gender parity indexes) (Table 5). It should be noted, however, that this adult model only explained about 31% of the variance in adulthood NCD suggesting that many other proximal factors may be at play (e.g. lifestyle - smoking, drinking, drug use, diet) which were not captured in our models. The higher % variance explained in the adolescent model vs adult model suggests NCDs in adolescence are particularly vulnerable to broader macro and societal factors that are evaluated in this analysis.

**Table 5:** Hierarchical bivariate and multivariable determinants of NCD DALYs among adults in 194 countries, 2015

Domain/Indicator		Outcome: DALYs attributed to NCDs among adults (rate, 2015)			
		Bivariate			Multivariable
		R-Square	Β estimate (Standard error) *P*-value	StB	Β estimate (Standard error) *P*-value
**Level 3 Model- Distal: Macro Structural Factors** ^**a**^					
**Conflict**	**Source & Year**				
Battle related deaths (total; log)	World Bank, 2013	9%	492.95 (242.94) 0.0487	0.17	–
Internally displaced persons (total; log)	World Bank, 2013	0%	31.75 (218.61) 0.8847	0.01	–
Refugee populations by country of asylum (total; log)	World Bank, 2014	3%	−262.897 (127.60) 0.0410	- 0.13	
**Governance**					
Political stability/ absence of terrorism	World Bank, 2013	2%	− 960.160 (512.51) 0.0673	−0.13	–
Government effectiveness ^1^	World Bank, 2013	7%	− 1831.88 (498.67) 0.0003	−0.26	–
Corruption ^1^	World Bank, 2013	4%	− 1288.79 (507.14) 0.0119	−0.18	−1288.79 (507.14) 0.0119
**Environment**					
Frequency of natural disasters (total; log)		0.3%	−487.76 (751.42) 0.5172	−0.05	–
Cost damage of natural disasters (USD; log)		0.8%	− 257.35 (403.53) 0.5266	−0.09	–
					**Distal Model R2:** **4%**
**Level 2 Model- Intermediate I: National Structural Factors** ^**b**^					
**Infrastructure and Urbanization**					
Urbanization (% of population; log)	UNICEF, 2016	6%	− 3405.14 (1004.45) .0009	−0.24	− 2668.44 (1064.88) 0.0131
Access to electricity (% population; cubed)	World Bank	5%	−0.0037 (0.0012) <.0001	−0.22	0.0025 (0.0015) 0.1040
**Population Density**					
Population density (people per m^2^ land; log)	World Bank	0.8%	− 445.2 (378.10) 0.2406	−0.09	–
**Telecommunications Access**					
Mobile cellular subscriptions (per 100 people; log)	World Bank, 2014	15%	− 5158.49 (919.96) <.0001	−0.38	–
Internet users (per 100 people; log)	World Bank, 2014	5%	− 1098.425 (376.30) 0.0040	−0.17	–
**National Wealth**					
GDP per capita, 5 year lag (USD; log) ^2^	World Bank, 2014	9%	− 1168.48 (271.40) <.0001	−0.25	− 1515.97 (586.38) 0.0105
**Health Spending**					
Government expenditure on health, 2 year lag (% of total health expenditure; log)	WHO, 2013	4%	− 2627.68 (1032.80) 0.0118	−0.15	–
Total health expenditure per capita, 2 year lag (PPP, NCU per USD; log)^2^	WHO, 2013	8%	−999.81 (250.55) <.0001	−0.23	–
					**Intermediate I Model R2:** **14%**
**Level 3- Intermediate II: Community, Household & Individual Factors** ^**c**^					
**Socioeconomic Status (Education and Employment)**					
Adult literacy rate (% of adults ages 15+ years; cubed)	World Bank	0.7%	−0.0015 (0.0016) 0.3635	−0.07	–
Primary school enrolment ratio (gross %; log)	World Bank, 2003–2014	0.1%	1311.79 (3489.26) 0.7074	0.02	–
Secondary school enrolment ratio (gross %; log)	UNICEF, 2013	1%	− 1465.82 (990.54) 0.1410	−0.09	4166.21 (2642.13) 0.1182
Employment to population ratio (% of adults 15+ years; log)	World Bank	0.2%	1325.91 (2539.92) 0.6023	0.04	
**Youth Empowerment**					
Youth literacy rate (% total 15–24 year olds; squared)^4^	World Bank, 2014	2%	−0.3266 (0.2154) 0.1320	−0.11	–
Female youth literacy rate (% 15–24 year olds; squared)^4^	World Bank, 2014	1%	−0.2504 (0.1997) 0.2125	− 0.09	–
Youth unemployment rate (% total 15–24 year olds; log)	World Bank, 2013	0%	1.5364 (672.15) 0.9982	0.0002	–
Adolescent fertility rate (births per 1000 females aged 15–19 years; log)	World Bank, 2009–2014	0%	−81.08 (485.13) 0.8675	−0.012	–
**Women Empowerment and Gender Equity**					
Total fertility rate (births per woman; log)	World Bank, 2013	0.7%	1223.67 (1106.03) 0.2700	0.08	–
Adult female literacy rate (% females 15+ years who can read and write; cubed)	World Bank	0.3%	−0.0010 (0.0015) 0.5232	−0.05	–
Women in parliament (% of parliamentary seats held by women; log)	World Bank, 2014	2%	− 1270.29 (714.36) 0.0771	−0.13	–
Secondary school gender parity index (ratio of girls to boys in secondary education; log)	World Bank, 2013	2%	− 5512.67 (2829.38) 0.0532	−0.12	−18,846.97 (5432.46) 0.0008
Tertiary school gender parity index (ratio of girls to boys in tertiary education; log)	World Bank, 2013	2%	− 1268.27 (834.61) 0.1308	−0.09	3176.62 (1776.29) 0.0770
**Income Equity**					
GINI index (log)	World Bank, 2012	0%	312.54 (2260.12) 0.8903	0.01	–
**Access to Health Services and Commodities**					
Out of pocket expenditure as % of total health expenditure (log)	WHO, 2013	0.5%	− 513.4 (524.81) 0.3292	−0.07	–
Physician density per 1000 population (log)	WHO, 2003–2013	3%	− 715.62 (366.23) 0.0529	− 0.15	1786.88 (844.88) 0.0371
					**Intermediate II Model R2:** **31%**

Note: variables significant at *p* < 0.20 in bivariate analysis were entered into elastic net linear regression models; ^1^ Political stability and Corruption were strongly collinear (> 80%) and thus only the later was entered into multivariable modeling; ^2^ Health expenditure per capita and GDP per capita were strongly collinear (> 80%) and thus only the latter was entered into multivariable modeling; ^3^ Due to small sample size (*n* = 117 countries), GINI index not considered in multivariable analysis; ^4^ Female Youth Literacy Rate and Youth Literacy Rate Both were strongly collinear (> 80%) and thus only the latter was entered into multivariable modeling

^a^ Level 3 multivariable model includes all statistically significant (*p* < 0.15) distal variables as listed

^b^ Level 2 multivariable model includes level 3 model+ all statistically significant (*p* < 0.15) intermediate I variables as listed

^c^ Level 1 multivariable model includes level 2 model+ all statistically significant (*p* < 0.15) intermediate II variables as list

### National Policies, Laws and Legislations for NCD prevention

**Fig. 2:**
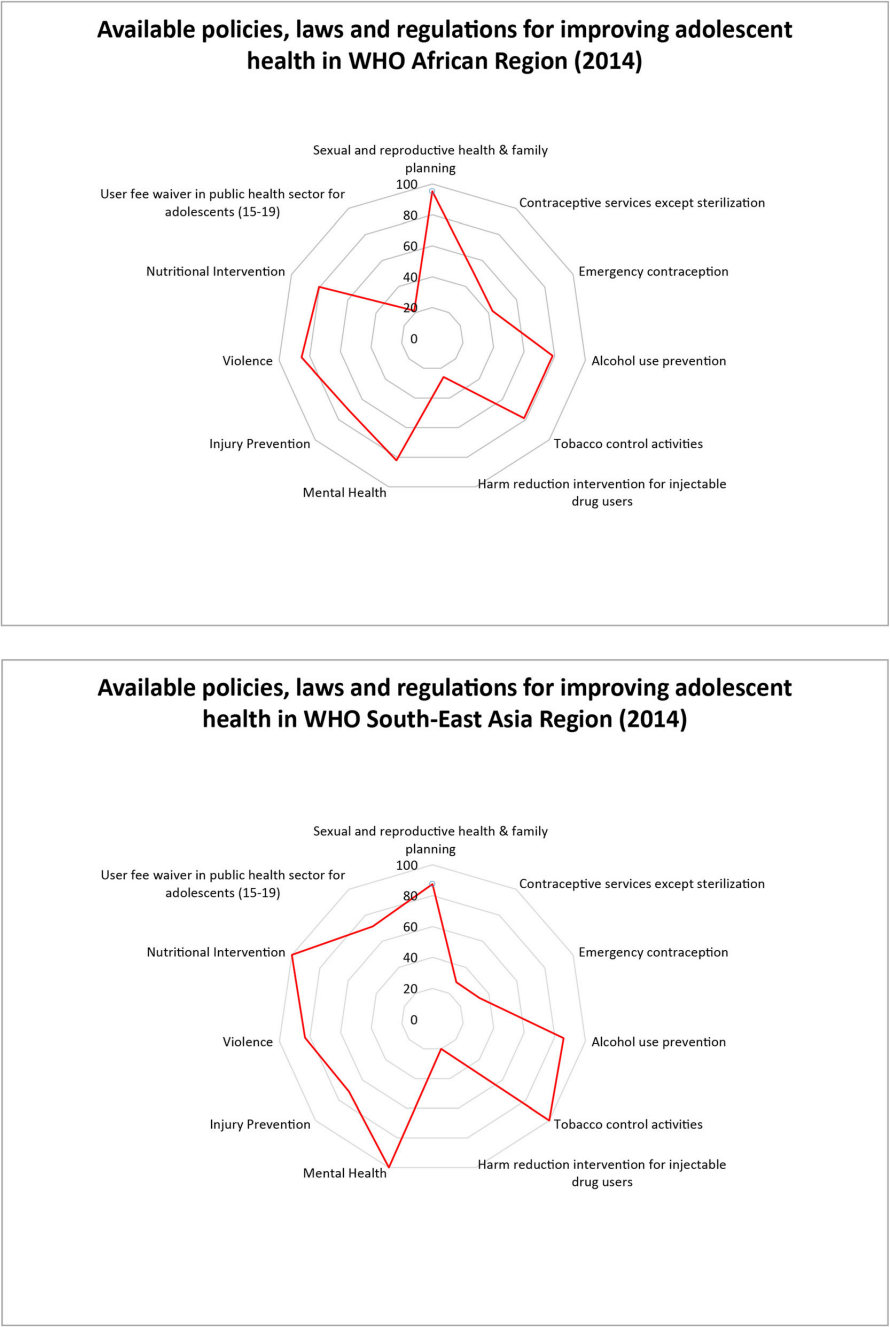
Policies, laws and regulations for adolescent NCD prevention in Africa and South East Asia

The availability of policies and laws targeting NCD-related lifestyle and behavioral risk factors among adolescents varied substantially across regions (Additional file 1). Across countries in the African region, the majority perform well with available national policies and strategies for sexual/reproductive health/family planning (95%), violence (85%) and mental health (82%), nutritional interventions (80%), alcohol use prevention (79%), tobacco control activities (79%), and injury prevention (71%). Similar patterns are observed in the South–East Asian region. Both regions are lacking in policies where laws and regulations allow minors to seek contraceptive services and emergency contraception without parental/caregiver consent and seek harm reduction interventions for injectable drug use. Policies which exempt adolescents aged 15–19 years from user fees in the public sector are more common in South-East Asia (71% of countries) and are lacking in Africa (21%) (Fig. 2). Across other regions (Additional file 1), the Western Pacific region specifically lacked in policies on mental health and nutritional interventions. In the Eastern Mediterranean, less than 45% of countries had adolescent-specific policies on alcohol use and injury/violence prevention. Among Eastern Mediterranean region countries with available data, none had available national laws and regulations permitting adolescents to seek contraceptive services or emergency contraception, and for harm reduction interventions for illicit drug users. In fact, these were lacking in all regions. However, not all countries in these regions participated in the surveys, and sample sizes vary by region and within region by policy type.

### Evidence-based interventions and delivery platforms for NCD prevention

Evidence-based interventions for adolescent populations that specifically target their modifiable risk factors for NCDs in adolescent and later life, can be broadly categorized into those delivered through community-based, school-based, peer-based, and family-based platforms. Varying in their degrees of effectiveness and implementation in high-income countries (HICs) versus low-and-middle income countries (LMICs), these interventions have been used to address NCD risk factors and promote protective factors among adolescents. We detail effective interventions for NCD prevention and reduction among adolescents [11, 42–108], and provide a project-specific overview in the appendix (Additional file 1).

Implementing evidence-based initiatives as part of a multi-level, multi-component and inter-sectoral approach can enhance their effectiveness by providing holistic and sustainable solutions [12]. For instance, interventions incorporating policy measures, environmental changes for promoting physical activity, and education on healthy diet and physical education simultaneously have been more effective in addressing obesity among adolescents compared to individual interventions [109]. Another example is cash incentive programs, which seek to improve school retention, while addressing issues of poverty, by providing payment incentives to students and their parents. These programs have been effective in improving educational attainment and decreased aggression, crime rates, alcohol and tobacco use, unwanted pregnancies, and mental health symptoms and disorders. Moreover, these have achieved long-term impact post-intervention, that last between 1 and 15 years [105, 110–112]. Lastly, mass media and social marketing interventions that target adolescents, their families, and broader communities, have been effective in altering social norms and have served as a platform for grassroots movements by empowering adolescents and broader communities [12, 113]. Recent advances in digital technology such as widespread availability and use of mobile phones, social media and online games may also serve as valuable intervention platforms for adolescents. Overall, efforts aimed at improving NCDs among adolescents and in later life should consider taking on holistic solutions that seek to build capacity within individual sectors while enhancing coordination across sectors.

## Discussion

We conducted a comprehensive review of NCD burden and determinants among adolescents, and also derived an evidence-based conceptual framework for tracking pathways to NCD development. We noted that NCDs are a prevailing public health concern among adolescents globally, among which, mental health conditions including depression and conduct disorders are leading disorders among both males and females. Proximal behavioral and lifestyle risk factors are important to disease burden among adolescents, and our analyses show that much of the burden of NCDs in adulthood are related to these modifiable factors that start in adolescence. Most notably, poor diets, smoking, alcohol use, drug use, and indicators of metabolic syndrome (high blood pressure, high cholesterol, diabetes) are the largest contributors to adulthood NCDs and the risks of each of these typically begin in adolescence. Our statistical model also demonstrates that these proximal factors are influenced by broader community and macro factors including social equality, youth empowerment, economic growth and health expenditure, infrastructure and state development, and good governance and security/stability. We also found that, globally, there is momentum towards implementing NCD specific policies/laws/legislations at the national level, but that there is a general lack of policies and laws allowing minors to seek contraceptive services and emergency contraception without consent, and to seek harm reduction interventions for injectable drug use. Additionally, mental health and nutrition related policies exist only variably across geographic regions.

Another consideration are the commercial determinants of health, which refer to consumption of commercial products such as processed food, tobacco, and alcohol, and the vested interests of corporations to encourage their use [114]. The commercial drivers of ill health are related to the lifestyle risk factors presented in our work as the tobacco, alcohol, and processed food and beverage industries influence consumption of products related to poor diets, smoking, and alcohol use, which in turn impact adolescent NCD burden [6, 115]. The global food industry has been identified as the leading driver of NCD epidemics related to diet [116]. The commercial determinants of health fall under the larger macro, societal and political social determinants of NCDs in adolescents, and they modify the behaviours of individuals in meaningful ways.

Our conceptual framework allows for the visualization of the diverse and complex web of pathways that shape NCDs among young people and in later life. In addition, it identifies critical windows or time periods for intervention, as well as the types of interventions that are necessary to mitigate and address the current and future burden. This conceptual framework could also be used to guide and strengthen the monitoring and evaluation of NCDs among adolescents that is urgently needed. Research on NCDs among adolescents has substantially focused on their engagement in key risk and protective behaviours [5, 12]. However, this study emphasizes that a paradigm shift is needed in order to recognize the critical role of underlying structural and societal determinants of health on NCDs among adolescents.

Key global and regional trends among adolescents highlight the significant burden of NCDs with a particular emphasis on mental illnesses such as conduct and major depressive disorders. This burden persists despite many countries globally, including 82% of countries in the African region, reporting the introduction of mental health policies. These trends may indicate weak implementation of key policies, funding, and political will to address NCDs among young people in many LMICs.

Risk and protective factors for NCDs among adolescents, including tobacco smoking, use of alcohol, physical inactivity, unemployment, and overweight, vary widely by region and sex. These findings align with previous empirical research on the burden of disease, risk and health behaviours among adolescent populations [5]. It also emphasizes the need to reduce the current burden of NCDs among young people, and prevent the heavy adult mortality and morbidity burden associated with risk factors for NCDs acquired in early life [5]. The diverse trends and inequities highlight the need to develop regional- and country-specific policies and programs to target key contributors to the NCD burden according to need.

Macro and societal factors across the life-course play a critical role on the burden of current and future NCDs among young people. Basic security, humanitarian issues and effective governance represent significant underlying structural determinants that shape both intermediate and proximal factors. Furthermore, infrastructure (e.g., urbanization and access to electricity), access to resources, health spending, human resources for health, gender equity and youth empowerment represent key intermediary influences that contribute significantly to NCDs among adolescents. These findings challenge the dominant perspective that individual and lifestyle risk and protective factors represent the primary contributors to NCDs, and emphasize the importance of underlying macro-level determinants. Other studies have also noted that broader social and economic determinants of health are linked to mortality, morbidity, and risk and protective factors among adolescents [12]. A paradigm shift in the conceptualization of NCDs and risk factors is therefore critical to developing effective interventions and policies to prevent and mitigate the increasing burden among adolescents. Investments in and emphasis on using multi-level and cross-sectoral interventions and policies that address these diverse influences must be prioritized by countries globally to achieve improvements in NCDs among adolescents. For example, current and future efforts to reduce the risk factors associated with NCDs could be focused on developing primary health-care hubs at the lowest possible level of the health-care system with essential infrastructure and human resources [6]. Health services that include NCD management and screening in community health units in villages have reported benefits in Ethiopia, Malawi, Namibia, Rwanda, and Uganda [117, 118]. These initiatives have trained community health workers to deliver integrated programs for multiple conditions at the community level and to address the needs of women and children which has resulted in improved health outcomes [117, 118]. Another focal point in the efforts to combat NCDs is the synergistic effort to reduce health inequalities and improve the equity of government health expenditure by financing universal health coverage [119]. Rwanda has used funding from HIV programs to expand health insurance coverage for poor sections of the population to improve access to health services, including those for NCDs [119]. While there is limited information to differentiate between the initiatives focused on adolescent or adult populations specifically, our results are congruent with the current focus on reducing NCD rates by means of supporting the improvement of state governance and increasing health expenditure to decrease health, gender, and socioeconomic inequalities. Urbanization and improved country development are suggestive of overall economic development and can be signals of improved access to health services for the management and treatment of NCDs.

Our work found that policies/laws/legislations targeting NCD risk factors among adolescents generally appear to exist in many countries, but actual implementation and impact data on these NCD policies and laws is limited. Although the MNCAH policy indicator dashboard provides information on the availability of policies or laws on NCDs by region and country, it lacks detailed information on implementation and monitoring and evaluation for impact. There is a strong need for the latter in particular as it’s needed to ensure and improve the quality and sustainability of programs, interventions, and policies targeting NCD risk factors. Tracking and holding countries accountable to their commitments for preventing NCDs is also critical, and can be initiated through preparing a core set of NCD monitoring indicators that can be used to evaluate and inform programs and policies that target NCD risk factors and health outcomes.

Our study is unique in that it used robust statistical modelling methods to explore the contributions of underlying distal, intermediate and proximal determinants in shaping the burden of NCDs among young people and adults. However, a few limitations should be noted. Firstly, our analysis was ecological (country-level) and thus results may be prone to the ecological fallacy. The sample size (*n* = 194) was not large but our final models were sufficiently powered with the number of covariates included. Our cross-sectional analysis could not infer temporality and associations do not necessarily suggest causality. Several important indicators (e.g. information on peer, family, and community social support systems, interpersonal relationships) and nutrient deficiency (e.g. anemia) and dietary intake did not have adequate available data for analysis and thus their effect should not be understated. For instance, while IHME databases have indicators on prevalence/incidence for dietary iron deficiency and hemoglobinopathies and hemolytic anemias for adolescents, estimates for many LMICs is missing or incomplete, and therefore we could not use this data in our analysis. Moreover, data used in this study are from publicly available sources such as the Global Health Observatory Data Repository and the World Bank database, and thus analyses and inference are limited to the quality of these data repositories. These sources often rely on survey datasets and other administrative sources that have known challenges in LMICs [120], and estimates may be modeled or direct estimates. However these estimates are amongst the best available globally and inferences will be meaningful, nonetheless must be interpreted with caution. The WHO MNCAH policy dashboard collects data only on existence of policies and thus the analysis in this study may not be representative of the country’s actual policy implementation impact.

Efforts to prevent the burden of NCDs among adolescents and in later adult life represents an area that necessitates further research, investment and intervention. Key stakeholders, including State and non-State actors, working to improve adolescent health should address under-researched and under-funded areas that represent critical determinants of NCD burden and illness in many LMICs [106]. Evidence from this study can be used to re-frame the current situation of NCDs in adolescents, highlighting their pathways/determinants and existence (or lack thereof) of interventions/strategies for countering the diseases. Our findings can be used by UN bodies, government/policy-makers, development partners, and academia to target areas of concern for intervention and for identifying future research priorities. The critical role of structural and societal determinants on NCDs in this population must be recognized, including national governance and youth empowerment. Efforts to address these underlying influences require interventions and policies that span multiple sectors and determinants.

## Conclusions

The findings of this study demonstrate the importance of adopting a more holistic approach to the prevention and reduction of NCD burden among adolescents, globally. The development and implementation of this approach requires a multilevel design that applies a life course perspective and addresses determinants across individual, community, national and societal levels. In addition, cross-sectoral collaboration is critical to ensuring effective development and implementation of policies to prevent NCDs among young people.

## Supplementary Information

**Additional file 1: Appendix 1**: Hierarchical levels, domains, and indicators related to NCDs among adolescents and later adulthood used for multivariable statistical modeling. **Appendix 2**: Top NCD DALYs in adolescents across WHO regions. **Appendix 3**: Youth and adulthood DALYS related to NCD risk factors that begin in adolescence. **Appendix 4**: Top NCD DALYS for health outcomes in adults aged 15–49 and 50–69 years. **Appendix 5**: Policies, laws and regulations for adolescent NCD prevention in the Eastern Mediterranean, the Americas, Europe, and the Western Pacific. **Appendix 6**: Policies, laws and regulations indicator breakdown for all regions by country. **Appendix 7**: Evidence-based NCD health interventions for adolescents and lifestyle factors among adolescents to prevent adulthood NCDs.

## Data Availability

The datasets analyzed during the current study are available in the Global Health Observatory Data Repository, https://www.who.int/gho/en/, the State of the World’s Children global statistics database, https://data.unicef.org/resources/state-worlds-children-2017-statistical-tables/, the World Bank database, https://data.worldbank.org/, the United Nations Statistics Division,http://www.un.org/en/development/desa/publications/world-population-prospects-2015-revision.html, and the 2015Global Burden of Disease (GBD) study housed at the Institute for Health Metrics and Evaluation (IHME), http://www.healthdata.org/gbd.

## Abbreviations

DALY: Disability-adjusted life years
GBD: Global burden of disease
IHME: Institute for health metrics and evaluation
LMIC: Low and middle income country
MNCAH: Maternal, newborn, child, and adolescent health
NCD: Non-communicable disease
