# Supplementary material for: Non-communicable diseases among adolescents: current status, determinants, interventions and policies
Source: BMC Public Health. 2020 Dec 14;20:1908. doi: 10.1186/s12889-020-09988-5 (PMC7734741; doi:10.1186/s12889-020-09988-5)
Supplement: Supplementary file 1 — Additional file 1: Appendix 1: Hierarchical levels, domains, and indicators related to NCDs among adolescents and later adulthood used for multivariable statistical modeling. Appendix 2: Top NCD DALYs in adolescents across WHO regions. Appendix 3: Youth and adulthood DALYS related to NCD risk factors that begin in adolescence. Appendix 4: Top NCD DALYS for health outcomes in adults aged 15–49 and 50–69 years. Appendix 5: Policies, laws and regulations for adolescent NCD prevention in the Eastern Mediterranean, the Americas, Europe, and the Western Pacific. Appendix 6: Policies, laws and regulations indicator breakdown for all regions by country. Appendix 7: Evidence-based NCD health interventions for adolescents and lifestyle factors among adolescents to prevent adulthood NCDs. [file 12889_2020_9988_MOESM1_ESM.docx]

**Appendices**

**Technical Appendix**:

*Literature review methods*

We searched PubMed, Google Scholar, WHO, UNICEF, UNFPA, and the NCD Alliance for articles published between Jan 1st, 1980 to Sept 30th, 2017. Reference lists of retrieved articles were further reviewed for additional relevant articles. Key search terms included, “adolescen*”, “youth”, “young people”, “health” AND “risk factors” or “protective factor” or “determinant” or “issue” or “policies” or “interventions” or “structural” or “community-based” or “school-based” or “peer-based” or “family-based” or “cross-cutting” or “conceptual frameworks” or “conceptual models” AND “non-communicable diseases” or “NCD” AND “low-income” or “middle-income” or “LMIC” or “developing countr*.

*WHO Maternal, Newborn, Child and Adolescent Health (MNCAH) policy indicator database*

The WHO Maternal, Newborn, Child and Adolescent Health (MNCAH) policy indicator database contains data on eleven adolescent NCD-related risk and health outcome policies, laws and regulations from 104 low and middle income countries (LMICs) globally. We obtained this data and explored country-level readiness for NCD initiatives by using the suggested scoring system. Available responses in the dashboard were “Yes, No, Unknown/not reported, Not enquired” which were coded as “1, 0, missing, missing” to represent the existence and or non-existence of a policy, respectively. It should be noted that the WHO MNCAH dashboard collects data only on existence of policies, not their implementation. This scoring was used to calculate an individual indicator score, a composite score and percentage for eleven policy indicators. The composite percentage score was used to construct a radar plot of the availability of select policies for each of the regions.

**Appendix 1**: Hierarchical levels, domains, and indicators related to NCDs among adolescents and later adulthood used for multivariable statistical modeling


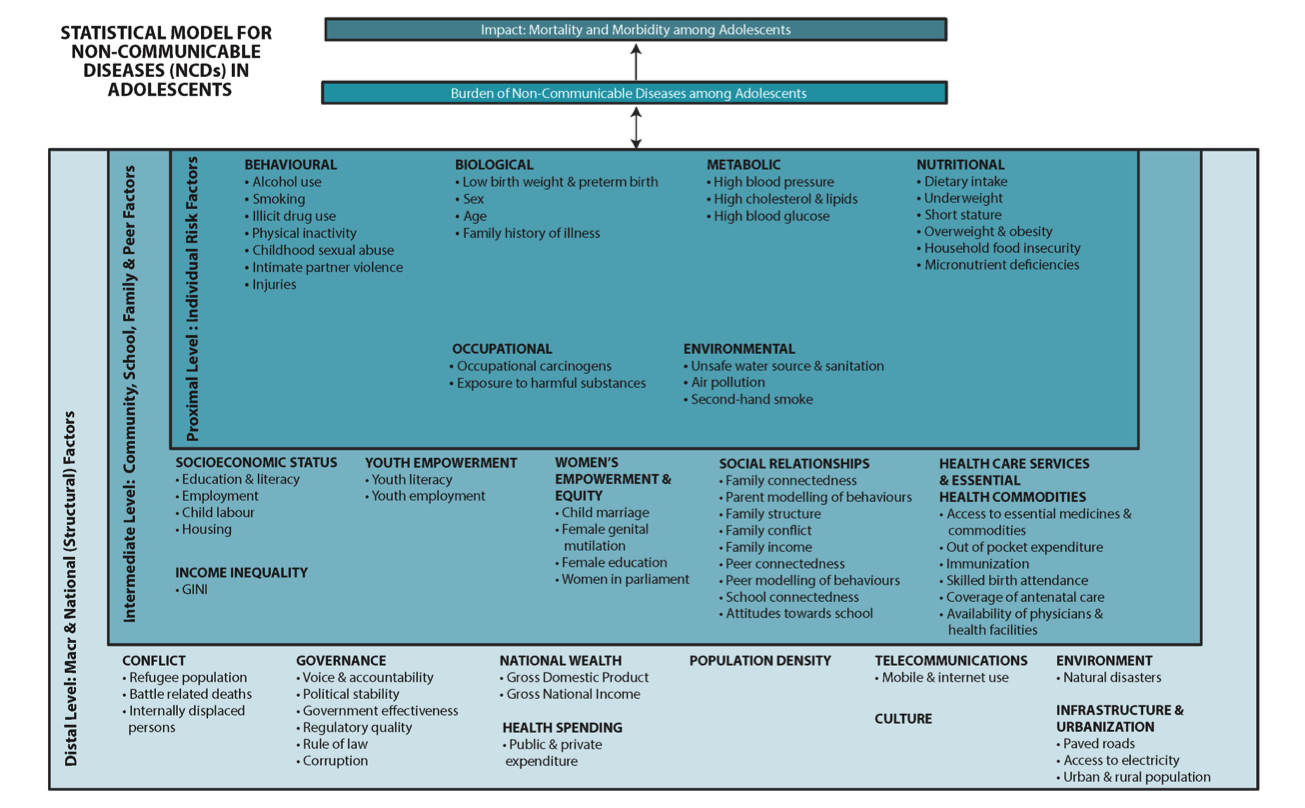
*Note: multivariable analysis focused on intermediate and distal level domains only.*

Table: Indicators, data source and final year collected for quantitative analysis

| **Indicator** | **Data Source** | **Year of Data Collection** |
| --- | --- | --- |
| Battle related deaths (total; log) | World Bank | 2013 |
| Internally displaced persons (total; log) | World Bank | 2013 |
| Refugee populations by country of asylum (total; log) | World Bank | 2014 |
| Political stability/absence of terrorism | World Bank | 2013 |
| Government effectiveness | World Bank | 2013 |
| Corruption | World Bank | 2013 |
| Frequency of natural disasters (total; log) | Emergency Events Database (EM-DAT), Centre for Research on the Epidemiology of Disasters (CRED) | 2015 |
| Cost damage of natural disasters (USD; log) | Emergency Events Database (EM-DAT), Centre for Research on the Epidemiology of Disasters (CRED) | 2015 |
| Urbanization (% of population; log) | UNICEF | 2015 |
| Access to electricity (% population; cubed) | World Bank | 2015 |
| Population density (people per m^2^ land; log) | World Bank | 2015 |
| Mobile cellular subscriptions (per 100 people; log) | World Bank | 2014 |
| Internet users (per 100 people; log) | World Bank | 2014 |
| GDP per capita, 5-year lag (USD; log) | World Bank | 2014 |
| Government expenditure on health, 2-year lag (% of total health expenditure; log) | WHO | 2013 |
| Total health expenditure per capita, 2-year lag (PPP, NCU per USD; log) | WHO | 2013 |
| Adult literacy rate (% of adults ages 15+ years; cubed) | World Bank | 2015 |
| Primary school enrolment ratio (gross %; log) | World Bank | 2003-2014 |
| Secondary school enrolment ratio (gross %; log) | World Bank | 2013 |
| Employment to population ratio (% of adults 15+ years; log) | World Bank | 2015 |
| Youth literacy rate (% total 15-24 year olds; squared) | World Bank | 2014 |
| Female youth literacy rate (% 15-24 year olds; squared) | World Bank | 2014 |
| Youth unemployment rate  (% total 15-24 year olds; log) | World Bank | 2013 |
| Adolescent fertility rate  (births per 1,000 females aged 15-19 years; log) | World Bank | 2009-2014 |
| Total fertility rate (births per woman; log) | World Bank | 2013 |
| Adult female literacy rate  (% females 15+ years who can read and write; cubed) | World Bank | 2015 |
| Women in parliament (% of parliamentary  seats held by women; log) | World Bank | 2014 |
| Secondary school gender parity index  (ratio of girls to boys in secondary education; log) | World Bank | 2013 |
| Tertiary school gender parity index  (ratio of girls to boys in tertiary education; log) | World Bank | 2013 |
| GINI index (log) | World Bank | 2012 |
| Out of pocket expenditure as % of total health expenditure (log) | WHO | 2013 |
| Physician density per 1,000 population (log) | WHO | 2003-2013 |

**Appendix 2**: Top NCD DALYs in adolescents across WHO regions

| **Adolescents: 10-14**  **years old** | **Males** | | | **Females** | | |
| --- | --- | --- | --- | --- | --- | --- |
|  | **Health Outcome** | **DALYs (1000s)** | **% of Total** | **Health Outcome** | **DALYs (1000s)** | **% of Total** |
| **Africa Region** | Conduct disorder | 315 | 8.37 | Asthma | 294 | 8.42 |
|  | Asthma | 281 | 7.47 | Anxiety disorders | 239 | 6.84 |
|  | Major depressive disorder | 178 | 4.72 | Migraine | 231 | 6.62 |
|  | Sickle cell disorders | 162 | 4.31 | Major depressive disorder | 208 | 5.95 |
|  | Age-related and other hearing loss | 161 | 4.28 | Conduct disorder | 202 | 5.78 |
|  | Anxiety disorders | 160 | 4.25 | Acne vulgaris | 160 | 4.58 |
|  | Acne vulgaris | 147 | 3.92 | Dermatitis | 149 | 4.27 |
|  | Low back pain | 137 | 3.63 | Sickle cell disorders | 145 | 4.15 |
|  | Migraine | 136 | 3.61 | Age-related and other hearing loss | 133 | 3.81 |
|  | Dermatitis | 126 | 3.36 | Low back pain | 103 | 2.96 |
|  | **Total DALYs** | 11,073 |  | **Total DALYs** | 9,732 |  |
|  | **NCD DALYs** | 3,759 |  | **NCD DALYs** | 3,491 |  |
|  | **Percent of total NCD burden in region age group** |  | **47.93** | **Percent of total NCD burden in region age group** |  | **53.37** |
| **Eastern Mediterranean Region** | Conduct disorder | 190 | 9.46 | Migraine | 161 | 8.09 |
|  | Asthma | 145 | 7.20 | Anxiety disorders | 149 | 7.48 |
|  | Anxiety disorders | 102 | 5.05 | Asthma | 137 | 6.86 |
|  | Migraine | 84 | 4.18 | Conduct disorder | 126 | 6.33 |
|  | Epilepsy | 83 | 4.12 | Major depressive disorder | 93 | 4.68 |
|  | Low back pain | 71 | 3.53 | Epilepsy | 80 | 4.01 |
|  | Major depressive disorder | 69 | 3.45 | Congenital heart anomalies | 76 | 3.80 |
|  | Acne vulgaris | 68 | 3.37 | Acne vulgaris | 69 | 3.48 |
|  | Thalassemias trait | 57 | 2.86 | Low back pain | 65 | 3.26 |
|  | Congenital heart anomalies | 57 | 2.84 | Dermatitis | 59 | 2.96 |
|  | **Total DALYs** | 4,318 |  | **Total DALYs** | 3,606 |  |
|  | **NCD DALYs** | 2,010 |  | **NCD DALYs** | 1,991 |  |
|  | **Percent of total NCD burden in region age group** |  | **46.07** | **Percent of total NCD burden in region age group** |  | **50.93** |
| **European Region** | Conduct disorder | 140 | 10.95 | Anxiety disorders | 128 | 10.32 |
|  | Asthma | 87 | 6.79 | Migraine | 120 | 9.65 |
|  | Migraine | 84 | 6.59 | Conduct disorder | 89 | 7.22 |
|  | Anxiety disorders | 79 | 6.16 | Asthma | 89 | 7.19 |
|  | Low back pain | 63 | 4.96 | Major depressive disorder | 75 | 6.09 |
|  | Acne vulgaris | 58 | 4.58 | Low back pain | 67 | 5.43 |
|  | Major depressive disorder | 57 | 4.48 | Acne vulgaris | 61 | 4.92 |
|  | Age-related and other hearing loss | 35 | 2.78 | Viral skin diseases | 41 | 3.30 |
|  | Autism | 35 | 2.78 | Dermatitis | 34 | 2.77 |
|  | Epilepsy | 35 | 2.71 | Epilepsy | 30 | 2.41 |
|  | **Total DALYs** | 2,021 |  | **Total DALYs** | 1,756 |  |
|  | **NCD DALYs** | 1,274 |  | **NCD DALYs** | 1,239 |  |
|  | **Percent of total NCD burden in region age group** |  | **52.79** | **Percent of total NCD burden in region age group** |  | **59.29** |
| **Region of the Americas** | Conduct disorder | 222 | 10.20 | Anxiety disorders | 204 | 9.46 |
|  | Asthma | 221 | 10.17 | Migraine | 192 | 8.91 |
|  | Anxiety disorders | 122 | 5.60 | Asthma | 191 | 8.84 |
|  | Migraine | 112 | 5.14 | Major depressive disorder | 148 | 6.86 |
|  | Major depressive disorder | 88 | 4.04 | Conduct disorder | 140 | 6.47 |
|  | Low back pain | 76 | 3.51 | Low back pain | 94 | 4.37 |
|  | Dermatitis | 72 | 3.33 | Dermatitis | 82 | 3.79 |
|  | Acne vulgaris | 72 | 3.33 | Acne vulgaris | 81 | 3.76 |
|  | Other congenital birth defects | 62 | 2.84 | Viral skin diseases | 69 | 3.18 |
|  | Age-related and other hearing loss | 59 | 2.72 | Other congenital birth defects | 53 | 2.44 |
|  | **Total DALYs** | 3,645 |  | **Total DALYs** | 3,108 |  |
|  | **NCD DALYs** | 2,173 |  | **NCD DALYs** | 2,156 |  |
|  | **Percent of total NCD burden in region age group** |  | **50.88** | **Percent of total NCD burden in region age group** |  | **58.07** |
| **South-East Asia Region** | Conduct disorder | 498 | 9.64 | Migraine | 446 | 9.61 |
|  | Asthma | 312 | 6.05 | Anxiety disorders | 309 | 6.65 |
|  | Migraine | 259 | 5.02 | Conduct disorder | 261 | 5.61 |
|  | Acne vulgaris | 218 | 4.23 | Asthma | 261 | 5.61 |
|  | Epilepsy | 215 | 4.17 | Acne vulgaris | 219 | 4.72 |
|  | Age-related and other hearing loss | 214 | 4.14 | Major depressive disorder | 199 | 4.28 |
|  | Anxiety disorders | 213 | 4.13 | Epilepsy | 184 | 3.96 |
|  | Low back pain | 197 | 3.81 | Age-related and other hearing loss | 178 | 3.83 |
|  | Major depressive disorder | 175 | 3.39 | Low back pain | 178 | 3.82 |
|  | Autism | 144 | 2.79 | Congenital heart anomalies | 150 | 3.22 |
|  | **Total DALYs** | 11,525 |  | **Total DALYs** | 9,629 |  |
|  | **NCD DALYs** | 5,162 |  | **NCD DALYs** | 4,647 |  |
|  | **Percent of total NCD burden in region age group** |  | **47.37** | **Percent of total NCD burden in region age group** |  | **51.31** |
| **Western Pacific Region** | Conduct disorder | 269 | 9.06 | Anxiety disorders | 228 | 8.86 |
|  | Anxiety disorders | 160 | 5.40 | Acne vulgaris | 159 | 6.18 |
|  | Asthma | 155 | 5.23 | Thalassemias trait | 144 | 5.60 |
|  | Acne vulgaris | 155 | 5.21 | Asthma | 142 | 5.53 |
|  | Thalassemias trait | 124 | 4.19 | Conduct disorder | 137 | 5.31 |
|  | Age-related and other hearing loss | 111 | 3.72 | Migraine | 120 | 4.67 |
|  | Low back pain | 103 | 3.46 | Low back pain | 111 | 4.32 |
|  | Congenital heart anomalies | 100 | 3.36 | Major depressive disorder | 100 | 3.87 |
|  | Acute lymphoid leukemia | 99 | 3.35 | Congenital heart anomalies | 89 | 3.45 |
|  | Migraine | 97 | 3.25 | Scabies | 85 | 3.30 |
|  | **Total DALYs** | 5,465 |  | **Total DALYs** | 3,965 |  |
|  | **NCD DALYs** | 2,968 |  | **NCD DALYs** | 2,573 |  |
|  | **Percent of total NCD burden in region age group** |  | **46.22** | **Percent of total NCD burden in region age group** |  | **51.10** |

| **Adolescents: 15-19**  **years old** | **Males** | | | **Females** | | |
| --- | --- | --- | --- | --- | --- | --- |
|  | **Health Outcome** | **DALYs (1000s)** | **% of Total** | **Health Outcome** | **DALYs (1000s)** | **% of Total** |
| **Africa Region** | Major depressive disorder | 394 | 8.91 | Major depressive disorder | 454 | 11.93 |
|  | Asthma | 209 | 4.72 | Migraine | 260 | 6.85 |
|  | Conduct disorder | 208 | 4.69 | Anxiety disorders | 251 | 6.60 |
|  | Low back pain | 204 | 4.62 | Acne vulgaris | 213 | 5.60 |
|  | Acne vulgaris | 194 | 4.39 | Asthma | 204 | 5.36 |
|  | Epilepsy | 189 | 4.27 | Low back pain | 158 | 4.16 |
|  | Anxiety disorders | 166 | 3.76 | Age-related and other hearing loss | 123 | 3.24 |
|  | Age-related and other hearing loss | 160 | 3.61 | Conduct disorder | 118 | 3.11 |
|  | Migraine | 151 | 3.41 | Epilepsy | 106 | 2.79 |
|  | Sickle cell disorders | 125 | 2.83 | Dermatitis | 100 | 2.62 |
|  | **Total DALYs** | 10,587 |  | **Total DALYs** | 9,193 |  |
|  | **NCD DALYs** | 4,424 |  | **NCD DALYs** | 3,801 |  |
|  | **Percent of total NCD burden in region age group** |  | **45.21** | **Percent of total NCD burden in region age group** |  | **52.27** |
| **Eastern Mediterranean Region** | Major depressive disorder | 163 | 6.46 | Migraine | 208 | 7.86 |
|  | Low back pain | 149 | 5.92 | Major depressive disorder | 208 | 7.86 |
|  | Conduct disorder | 141 | 5.58 | Anxiety disorders | 179 | 6.79 |
|  | Migraine | 122 | 4.86 | Low back pain | 125 | 4.73 |
|  | Anxiety disorders | 115 | 4.54 | Other musculoskeletal disorders | 103 | 3.90 |
|  | Asthma | 101 | 3.99 | Asthma | 99 | 3.75 |
|  | Acne vulgaris | 98 | 3.90 | Acne vulgaris | 99 | 3.73 |
|  | Other musculoskeletal disorders | 96 | 3.82 | Conduct disorder | 82 | 3.12 |
|  | Epilepsy | 80 | 3.19 | Epilepsy | 74 | 2.82 |
|  | Hemorrhagic stroke | 71 | 2.81 | Ischemic heart disease | 59 | 2.23 |
|  | **Total DALYs** | 6,084 |  | **Total DALYs** | 4,551 |  |
|  | **NCD DALYs** | 2,520 |  | **NCD DALYs** | 2,640 |  |
|  | **Percent of total NCD burden in region age group** |  | **45.07** | **Percent of total NCD burden in region age group** |  | **46.80** |
| **European Region** | Low back pain | 150 | 8.36 | Major depressive disorder | 183 | 9.84 |
|  | Major depressive disorder | 128 | 7.16 | Migraine | 169 | 9.10 |
|  | Conduct disorder | 115 | 6.41 | Anxiety disorders | 160 | 8.57 |
|  | Migraine | 106 | 5.92 | Low back pain | 152 | 8.17 |
|  | Acne vulgaris | 102 | 5.70 | Acne vulgaris | 103 | 5.56 |
|  | Anxiety disorders | 95 | 5.31 | Asthma | 84 | 4.52 |
|  | Asthma | 75 | 4.19 | Conduct disorder | 66 | 3.54 |
|  | Epilepsy | 45 | 2.49 | Other musculoskeletal disorders | 50 | 2.67 |
|  | Other musculoskeletal disorders | 42 | 2.36 | Bipolar disorder | 44 | 2.37 |
|  | Opioid use disorders | 39 | 2.19 | Neck pain | 40 | 2.17 |
|  | **Total DALYs** | 2,791 |  | **Total DALYs** | 2,383 |  |
|  | **NCD DALYs** | 1,790 |  | **NCD DALYs** | 1,861 |  |
|  | **Percent of total NCD burden in region age group** |  | **50.10** | **Percent of total NCD burden in region age group** |  | **56.50** |
| **Region of the Americas** | Major depressive disorder | 240 | 8.27 | Major depressive disorder | 409 | 12.80 |
|  | Conduct disorder | 182 | 6.26 | Anxiety disorders | 293 | 9.18 |
|  | Low back pain | 164 | 5.64 | Migraine | 266 | 8.32 |
|  | Anxiety disorders | 163 | 5.61 | Low back pain | 195 | 6.10 |
|  | Other musculoskeletal disorders | 160 | 5.50 | Other musculoskeletal disorders | 174 | 5.45 |
|  | Asthma | 145 | 5.00 | Asthma | 157 | 4.89 |
|  | Migraine | 132 | 4.53 | Acne vulgaris | 130 | 4.07 |
|  | Acne vulgaris | 123 | 4.22 | Conduct disorder | 102 | 3.19 |
|  | Bipolar disorder | 62 | 2.12 | Bipolar disorder | 73 | 2.29 |
|  | Age-related and other hearing loss | 61 | 2.10 | Dermatitis | 67 | 2.11 |
|  | **Total DALYs** | 5,711 |  | **Total DALYs** | 4,240 |  |
|  | **NCD DALYs** | 2,907 |  | **NCD DALYs** | 3,198 |  |
|  | **Percent of total NCD burden in region age group** |  | **49.26** | **Percent of total NCD burden in region age group** |  | **58.40** |
| **South-East Asia Region** | Major depressive disorder | 455 | 6.32 | Migraine | 574 | 8.37 |
|  | Low back pain | 446 | 6.19 | Major depressive disorder | 529 | 7.70 |
|  | Migraine | 383 | 5.32 | Other musculoskeletal disorders | 415 | 6.04 |
|  | Conduct disorder | 364 | 5.05 | Anxiety disorders | 364 | 5.31 |
|  | Other musculoskeletal disorders | 319 | 4.43 | Acne vulgaris | 315 | 4.59 |
|  | Acne vulgaris | 315 | 4.38 | Low back pain | 305 | 4.45 |
|  | Anxiety disorders | 261 | 3.62 | Asthma | 203 | 2.96 |
|  | Asthma | 233 | 3.23 | Age-related and other hearing loss | 177 | 2.57 |
|  | Epilepsy | 229 | 3.18 | Conduct disorder | 173 | 2.53 |
|  | Age-related and other hearing loss | 219 | 3.05 | Rheumatic heart disease | 158 | 2.30 |
|  | **Total DALYs** | 14,633 |  | **Total DALYs** | 13,659 |  |
|  | **NCD DALYs** | 7,206 |  | **NCD DALYs** | 6,863 |  |
|  | **Percent of total NCD burden in region age group** |  | **44.76** | **Percent of total NCD burden in region age group** |  | **46.82** |
| **Western Pacific Region** | Acne vulgaris | 227 | 5.90 | Major depressive disorder | 269 | 7.70 |
|  | Conduct disorder | 218 | 5.66 | Anxiety disorders | 263 | 7.51 |
|  | Low back pain | 205 | 5.34 | Other musculoskeletal disorders | 225 | 6.44 |
|  | Major depressive disorder | 204 | 5.29 | Acne vulgaris | 221 | 6.32 |
|  | Anxiety disorders | 188 | 4.90 | Low back pain | 214 | 6.13 |
|  | Other musculoskeletal disorders | 171 | 4.45 | Migraine | 191 | 5.46 |
|  | Migraine | 139 | 3.62 | Asthma | 115 | 3.30 |
|  | Asthma | 123 | 3.20 | Conduct disorder | 99 | 2.83 |
|  | Age-related and other hearing loss | 113 | 2.93 | Scabies | 95 | 2.71 |
|  | Scabies | 103 | 2.67 | Premenstrual syndrome | 84 | 2.40 |
|  | **Total DALYs** | 6,403 |  | **Total DALYs** | 4,688 |  |
|  | **NCD DALYs** | 3,848 |  | **NCD DALYs** | 3,497 |  |
|  | **Percent of total NCD burden in region age group** |  | **43.95** | **Percent of total NCD burden in region age group** |  | **50.80** |

**Appendix 3**: Youth and adulthood DALYS related to NCD risk factors that begin in adolescence

|  | |  | |  | |  | |  | |  |  | |  |  | |  |  | | |  |
| --- | --- | --- | --- | --- | --- | --- | --- | --- | --- | --- | --- | --- | --- | --- | --- | --- | --- | --- | --- | --- |
|  |  | | **Both** | | | | | | **Male** | | | | | | **Female** | | | | | |
|  |  | | **Risk Factor** | | **DALYs (1000s)** | | **% of Total** | | **Risk Factor** | | | **DALYs (1000s)** | **% of Total** | | **Risk Factor** | | | **DALYs (1000s)** | **% of Total** | |
| **15-49 Years Old** | **1** | | High Body Mass Index | | 37,304 | | 13.19 | | High systolic blood pressure | | | 25,057 | 14.16 | | High body-mass index | | | 15,460 | 14.60 | |
|  | **2** | | High Systolic Blood Pressure | | 36,247 | | 12.82 | | Smoking | | | 24,355 | 13.77 | | High systolic blood pressure | | | 11,190 | 10.57 | |
|  | **3** | | Smoking | | 29,251 | | 10.34 | | High body-mass index | | | 21,844 | 12.35 | | High fasting plasma glucose | | | 11,061 | 10.45 | |
|  | **4** | | High Fasting Plasma Glucose | | 28,112 | | 9.94 | | Alcohol use | | | 20,231 | 11.43 | | High total cholesterol | | | 6,640 | 6.27 | |
|  | **5** | | Alcohol Use | | 25,620 | | 9.06 | | High total cholesterol | | | 17,112 | 9.67 | | Drug use | | | 6,044 | 5.71 | |
|  | **6** | | High Total Cholesterol | | 23,752 | | 8.40 | | High fasting plasma glucose | | | 17,052 | 9.64 | | Diet low in whole grains | | | 6,043 | 5.71 | |
|  | **7** | | Drug Use | | 19,181 | | 6.78 | | Drug use | | | 13,137 | 7.42 | | Diet low in fruits | | | 5,818 | 5.50 | |
|  | **8** | | Diet low in fruits | | 17,412 | | 6.16 | | Ambient particulate matter pollution | | | 11,596 | 6.55 | | Impaired kidney function | | | 5,638 | 5.33 | |
|  | **9** | | Diet low in whole grains | | 17,409 | | 6.16 | | Diet low in fruits | | | 11,594 | 6.55 | | Alcohol use | | | 5,388 | 5.09 | |
|  | **10** | | Ambient particulate matter pollution | | 16,975 | | 6.00 | | Diet low in whole grains | | | 11,366 | 6.42 | | Ambient particulate matter pollution | | | 5,379 | 5.08 | |
|  |  | | Top 10 Total DALYs (1000s) | | 251,261 | |  | | Top 10 Total DALYs (1000s) | | | 173,343 |  | | Top 10 Total DALYs (1000s) | | | 78,660 |  | |
|  |  | | Total DALYs (Level 0) (1000s) | | 282,806 | |  | | Total DALYs (Level 0) (1000s) | | | 176,928 |  | | Total DALYs (Level 0) (1000s) | | | 105,878 |  | |
|  |  | | **Risk Factor** | | **DALYs (1000s)** | | **% of Total** | | **Risk Factor** | | | **DALYs (1000s)** | **% of Total** | | **Risk Factor** | | | **DALYs (1000s)** | **% of Total** | |
| **50-69 Years Old** | **1** | | High systolic blood pressure | | 99,826 | | 30.66 | | Smoking | | | 62,298 | 31.03 | | High systolic blood pressure | | | 38,801 | 31.10 | |
|  | **2** | | Smoking | | 76,743 | | 23.57 | | High systolic blood pressure | | | 61,025 | 30.39 | | High body-mass index | | | 32,022 | 25.66 | |
|  | **3** | | High body-mass index | | 67,911 | | 20.86 | | High fasting plasma glucose | | | 38,329 | 19.09 | | High fasting plasma glucose | | | 28,328 | 22.70 | |
|  | **4** | | High fasting plasma glucose | | 66,656 | | 20.47 | | High body-mass index | | | 35,889 | 17.87 | | High total cholesterol | | | 16,443 | 13.18 | |
|  | **5** | | High total cholesterol | | 44,873 | | 13.78 | | High total cholesterol | | | 28,430 | 14.16 | | Smoking | | | 14,445 | 11.58 | |
|  | **6** | | Ambient particulate matter pollution | | 37,787 | | 11.61 | | Alcohol use | | | 24,618 | 12.26 | | Ambient particulate matter pollution | | | 13,498 | 10.82 | |
|  | **7** | | Alcohol use | | 30,571 | | 9.39 | | Ambient particulate matter pollution | | | 24,289 | 12.10 | | Diet low in whole grains | | | 11,619 | 9.31 | |
|  | **8** | | Diet low in fruits | | 29,982 | | 9.21 | | Diet low in fruits | | | 19,260 | 9.59 | | Diet low in fruits | | | 10,722 | 8.59 | |
|  | **9** | | Diet low in whole grains | | 29,622 | | 9.10 | | Diet low in whole grains | | | 18,003 | 8.97 | | Impaired kidney function | | | 10,524 | 8.43 | |
|  | **10** | | Impaired kidney function | | 25,479 | | 7.83 | | Diet high in sodium | | | 15,482 | 7.71 | | Household air pollution from solid fuels | | | 10,013 | 8.03 | |
|  |  | | Top 10 Total DALYs (1000s) | | 509,451 | | 156.49 | | Top 10 Total DALYs (1000s) | | | 327,623 |  | | Top 10 Total DALYs (1000s) | | | 186,415 |  | |
|  |  | | Total DALYs (Level 0) (1000s) | | 325,558 | |  | | Total DALYs (Level 0) (1000s) | | | 200,782 |  | | Total DALYs (Level 0) (1000s) | | | 124,776 |  | |

**Appendix 4**: Top NCD DALYS for health outcomes in adults aged 15-49 and 50-69 years

**Appendix 5**: Policies, laws and regulations for adolescent NCD prevention in the Eastern Mediterranean, the Americas, Europe, and the Western Pacific.


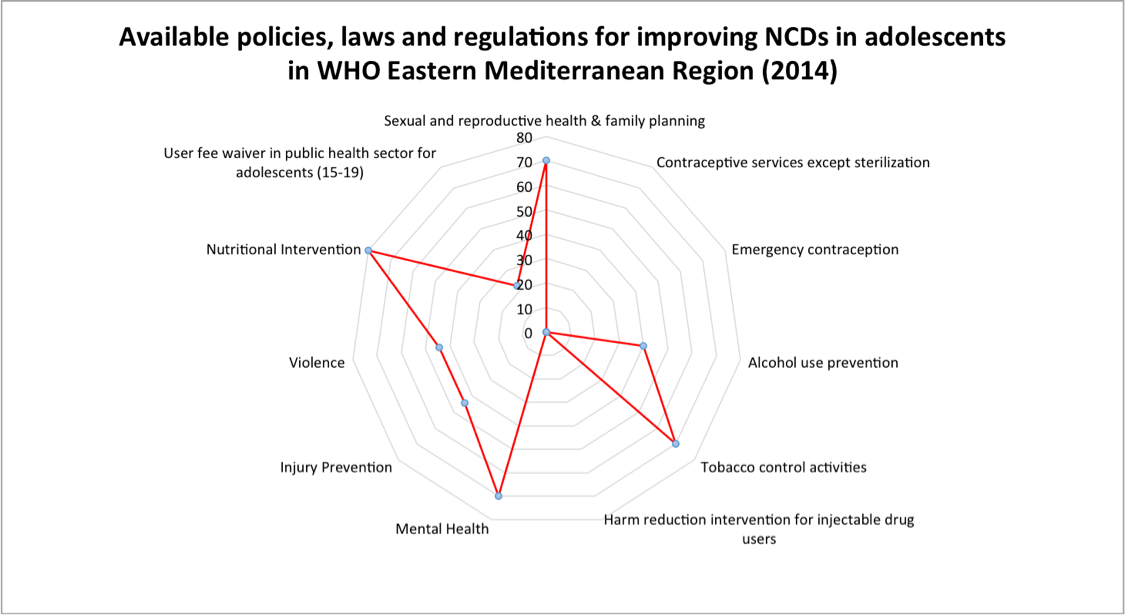


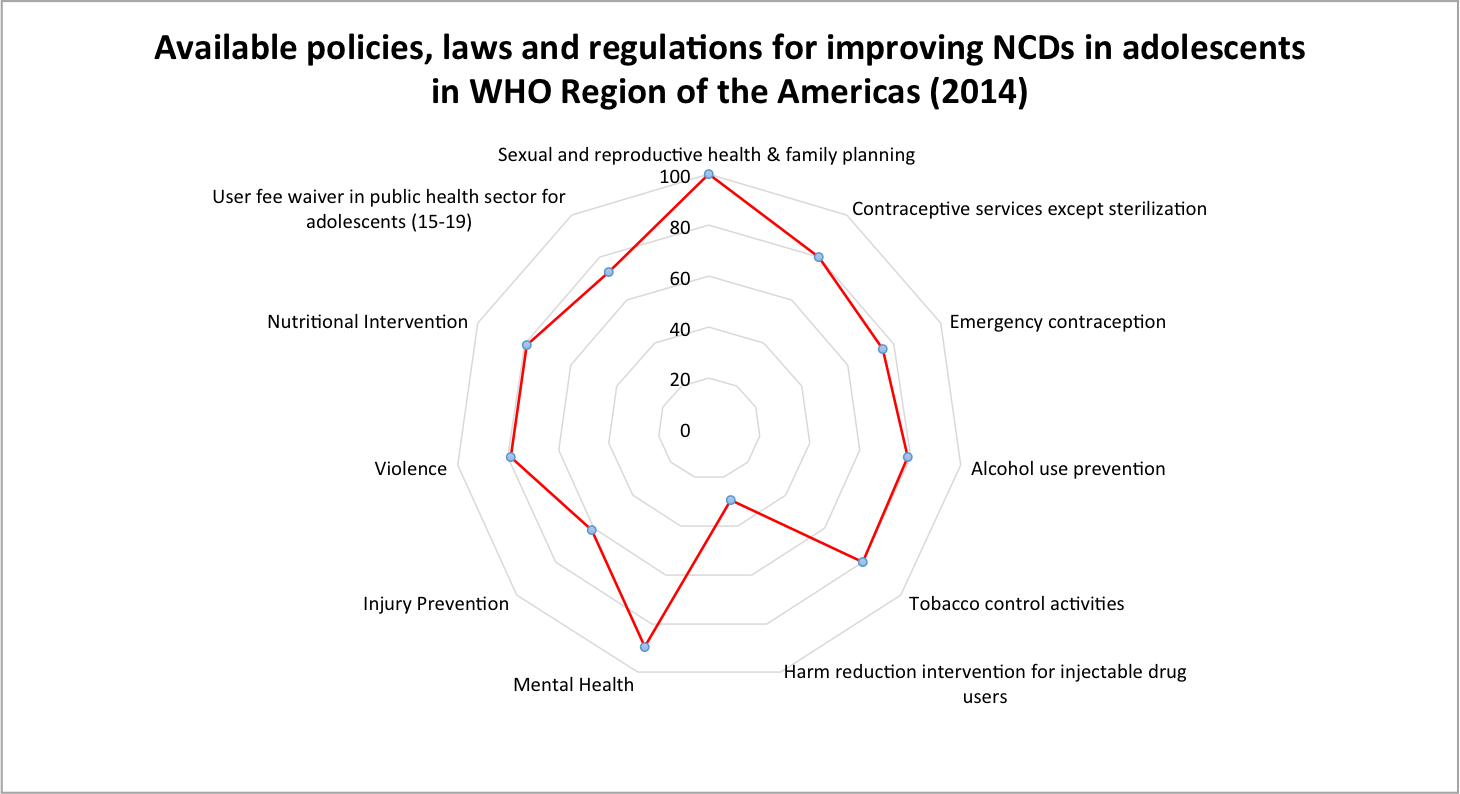


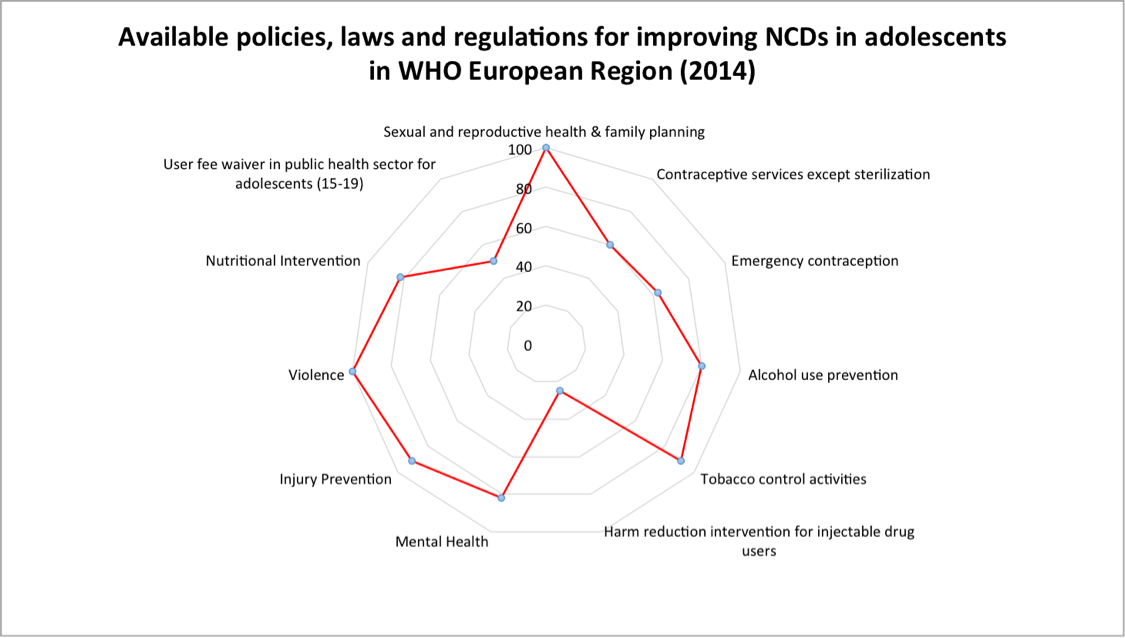


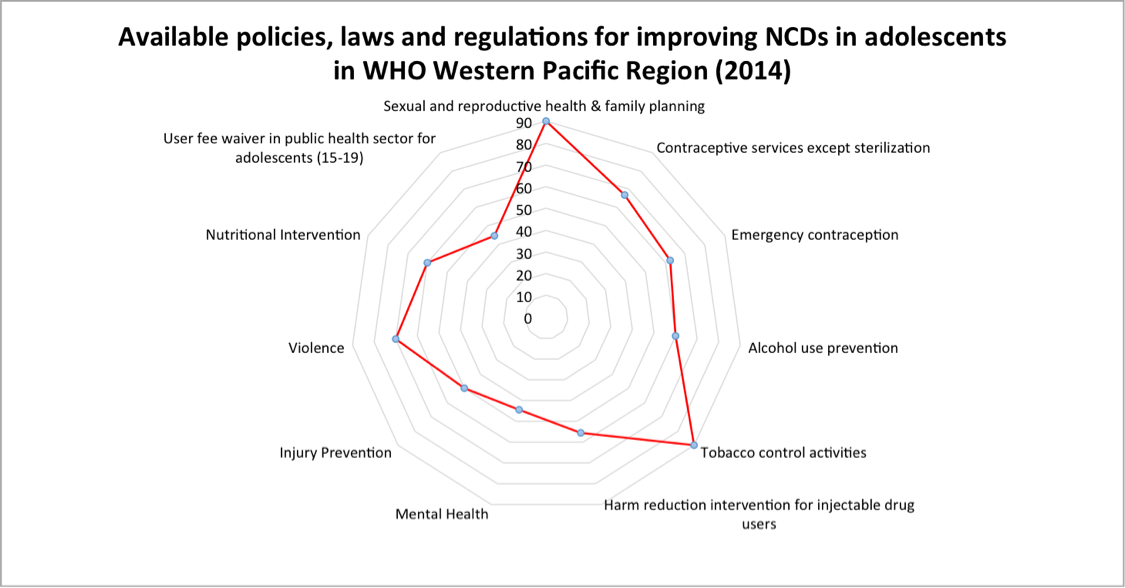


**Appendix 6**: Policies, laws and regulations indicator breakdown for all regions by country

| **WHO African Region** | | | | | | | |
| --- | --- | --- | --- | --- | --- | --- | --- |
| **Countries** | **National policies, strategies and plans for Adolescents** | | | | | | |
|  | **Sexual and reproductive health & family planning** | **Nutritional Intervention** | **Alcohol use prevention** | **Tobacco control activities** | **Mental health** | **Injury prevention** | **Violence** |
| 1. Angola | Yes | Yes | No | No | Unknown/not reported | No | Yes |
| 2. Benin | Yes | Yes | Yes | Yes | Yes | Unknown/not reported | Yes |
| 3. Botswana | Yes | No | Yes | Yes | No | Yes | Yes |
| 4. Burkina Faso | Yes | Yes | Yes | Yes | Yes | Yes | Yes |
| 5. Burundi | Yes | Yes | Yes | Yes | Yes | Yes | Yes |
| 6. Cameroon | No | No | No | No | No | No | No |
| 7. Central African Republic | Yes | Yes | Yes | Yes | Yes | Yes | Yes |
| 8. Chad | Unknown/not reported | Yes | Yes | Yes | Yes | Yes | Yes |
| 9. Comoros | Yes | Yes | Yes | Yes | Yes | Yes | Yes |
| 10. Congo | Yes | Yes | Yes | Yes | Yes | Yes | Yes |
| 11. Cote d'Ivoire | Yes | Yes | Yes | Yes | Yes | Yes | Yes |
| 12. Democratic Republic of the Congo | Yes | Yes | Yes | Yes | Unknown/not reported | Yes | Yes |
| 13. Equatorial Guinea | Yes | Unknown/not reported | Unknown/not reported | Unknown/not reported | Unknown/not reported | Unknown/not reported | Unknown/not reported |
| 14. Eritrea | Yes | No | No | No | No | No | No |
| 15. Ethiopia | Yes | Yes | No | No | Yes | Unknown/not reported | Yes |
| 16. Gabon | Yes | Yes | Yes | Yes | Yes | No | Yes |
| 17. Gambia | Yes | Yes | Yes | Yes | Yes | Yes | Yes |
| 18. Ghana | Yes | Yes | No | No | Yes | No | No |
| 19. Guinea | Yes | No | Yes | Yes | Yes | Yes | Yes |
| 20. Guinea-Bissau | Yes | Yes | Yes | Yes | Yes | No | Yes |
| 21. Kenya | Yes | Unknown/not reported | Yes | Yes | Unknown/not reported | Unknown/not reported | Unknown/not reported |
| 22. Lesotho | Yes | Yes | Yes | Yes | Yes | Yes | Yes |
| 23. Liberia | Yes | Yes | Yes | Yes | Yes | Yes | Yes |
| 24. Madagascar | Yes | No | No | No | No | No | Yes |
| 25. Malawi | Yes | Yes | Yes | Yes | Yes | Yes | Yes |
| 26. Mali | Yes | Yes | Yes | Yes | Yes | Yes | Yes |
| 27. Mauritania | Unknown/not reported | Unknown/not reported | Unknown/not reported | Unknown/not reported | Unknown/not reported | Unknown/not reported | Unknown/not reported |
| 28. Mozambique | Yes | Yes | Yes | Yes | Yes | Yes | Yes |
| 29. Namibia | Yes | No | No | No | No | No | No |
| 30. Niger | Yes | Yes | Yes | Yes | Yes | Yes | Yes |
| 31. Nigeria | Yes | Yes | Yes | Yes | Yes | Yes | Yes |
| 32. Rwanda | Yes | Yes | Yes | Yes | Yes | Yes | Yes |
| 33. Sao Tome and Principe | Yes | Yes | Yes | Yes | Yes | Yes | Yes |
| 34. Senegal | Yes | Yes | Yes | Yes | Yes | Yes | Yes |
| 35. Seychelles | Yes | Yes | Yes | Yes | Yes | Yes | Yes |
| 36. Sierra Leone | Yes | Yes | Yes | Yes | Yes | Yes | Yes |
| 37. South Africa | Yes | Yes | Yes | Yes | Yes | Yes | Yes |
| 38. South Sudan | No | No | No | No | No | No | No |
| 39. Swaziland | Yes | Yes | No | No | Yes | Unknown/not reported | Yes |
| 40. Togo | Yes | No | Yes | Yes | No | No | Yes |
| 41. Uganda | Yes | Yes | Yes | Yes | Yes | Yes | Yes |
| 42. United Republic of Tanzania | Yes | Yes | Yes | Yes | Yes | No | Yes |
| 43. Zambia | Yes | Yes | Yes | Yes | Yes | Yes | No |
| 44. Zimbabwe | Yes | Yes | Yes | Yes | Yes | Yes | Yes |
| **Total Yes** | **40** | **33** | **33** | **33** | **32** | **27** | **35** |
| **Total countries per policy indicator** | **42** | **41** | **42** | **42** | **39** | **38** | **41** |
| **Total %age** | **0.952** | **0.805** | **0.786** | **0.786** | **0.821** | **0.711** | **0.854** |

| **WHO African Region** | | | | |
| --- | --- | --- | --- | --- |
| **Countries** | **Laws and regulations allow minor adolescents to seek** | | | **User fee waiver in public health sector for adolescents (15-19 years)** |
|  | **Contraceptives services except sterilization** | **Emergency contraception** | **Harm reduction intervention for injectable drug users** |  |
| 1. Angola | No | No | No | Yes |
| 2. Benin | Yes | Yes | Yes | No |
| 3. Botswana | Yes | Yes | No | Yes |
| 4. Burkina Faso | Yes | Yes | Yes | No |
| 5. Burundi | No | No | No | No |
| 6. Cameroon | Unknown/not reported | Unknown/not reported | Unknown/not reported | No |
| 7. Central African Republic | Unknown/not reported | Unknown/not reported | Unknown/not reported | No |
| 8. Chad | Unknown/not reported | Unknown/not reported | No | No |
| 9. Comoros | No | No | No | No |
| 10. Congo | No | No | No | No |
| 11. Cote d'Ivoire | Yes | Yes | Yes | No |
| 12. Democratic Republic of the Congo | No | No | No | No |
| 13. Equatorial Guinea | Yes | Unknown/not reported | Unknown/not reported | No |
| 14. Eritrea | Yes | Yes | Unknown/not reported | No |
| 15. Ethiopia | Yes | Unknown/not reported | No | No |
| 16. Gabon | No | No | No | No |
| 17. Gambia | Yes | Yes | Unknown/not reported | No |
| 18. Ghana | Yes | Unknown/not reported | No | No |
| 19. Guinea | Yes | Yes | Unknown/not reported | No |
| 20. Guinea-Bissau | Unknown/not reported | Unknown/not reported | Unknown/not reported | No |
| 21. Kenya | Yes | Yes | Yes | Yes |
| 22. Lesotho | No | Unknown/not reported | Yes | No |
| 23. Liberia | No | No | No | Yes |
| 24. Madagascar | Unknown/not reported | No | No | No |
| 25. Malawi | Yes | Yes | Unknown/not reported | Yes |
| 26. Mali | Yes | Unknown/not reported | Unknown/not reported | No |
| 27. Mauritania | Unknown/not reported | Unknown/not reported | Unknown/not reported | No |
| 28. Mozambique | No | Unknown/not reported | No | No |
| 29. Namibia | No | No | No | No |
| 30. Niger | Yes | Yes | Yes | No |
| 31. Nigeria | Yes | Unknown/not reported | Unknown/not reported | No |
| 32. Rwanda | No | No | No | No |
| 33. Sao Tome and Principe | No | No | Unknown/not reported | No |
| 34. Senegal | Yes | Unknown/not reported | Unknown/not reported | No |
| 35. Seychelles | No | Not enquired | No | Not enquired |
| 36. Sierra Leone | No | No | No | Yes |
| 37. South Africa | Yes | Yes | Yes | Yes |
| 38. South Sudan | No | No | No | No |
| 39. Swaziland | No | No | No | No |
| 40. Togo | No | Unknown/not reported | No | No |
| 41. Uganda | Yes | Yes | No | Yes |
| 42. United Republic of Tanzania | Yes | Unknown/not reported | Yes | No |
| 43. Zambia | No | No | No | Yes |
| 44. Zimbabwe | No | No | No | Unknown/not reported |
| **Total Yes** | **19** | **12** | **8** | **9** |
| **Total countries per policy indicator** | **38** | **28** | **31** | **42** |
| **Total %age** | **0.500** | **0.429** | **0.258** | **0.214** |

Number of Countries included: 44

Number of Countries not included: 3 (Algeria, Cobo Verde, Mauritius)

Total number of Countries in the region: 47

| **WHO Region of the Americas** | | | | | | | |
| --- | --- | --- | --- | --- | --- | --- | --- |
| **Countries** | **National policies, strategies and plans for Adolescents** | | | | | | |
|  | **Sexual and reproductive health & family planning** | **Nutritional Intervention** | **Alcohol use prevention** | **Tobacco control activities** | **Mental health** | **Injury prevention** | **Violence** |
| 1. Argentina | Yes | Yes | Yes | Yes | Yes | No | Yes |
| 2. Belize | Yes | No | No | No | Yes | No | No |
| 3. Bolivia (Plurinational State of) | Yes | Yes | Yes | Yes | Yes | Yes | Yes |
| 4. Brazil | Yes | Yes | Yes | Yes | Yes | Yes | Yes |
| 5. Colombia | Yes | No | No | No | Yes | No | No |
| 6. Costa Rica | Yes | Yes | Yes | Yes | Yes | Yes | Yes |
| 7. Cuba | Yes | Yes | Yes | Yes | Yes | Yes | Yes |
| 8. Ecuador | Yes | Yes | Yes | Yes | Yes | No | Yes |
| 9. El Salvador | Yes | Yes | Yes | Yes | Yes | Yes | Yes |
| 10. Guatemala | Yes | Yes | Yes | Yes | Yes | Unknown/not reported | Yes |
| 11. Guyana | Yes | Yes | Yes | Yes | Yes | Yes | Yes |
| 12. Haiti | Yes | No | No | No | No | No | No |
| 13. Honduras | Yes | Yes | Yes | Yes | Yes | Yes | Yes |
| 14. Jamaica | Yes | Unknown/not reported | Unknown/not reported | Yes | Unknown/not reported | Unknown/not reported | Unknown/not reported |
| 15. Mexico | Yes | Yes | Yes | Yes | Yes | Yes | Yes |
| 16. Nicaragua | Yes | Yes | No | No | Yes | Yes | Yes |
| 17. Panama | Yes | Yes | Yes | Yes | Yes | Yes | Yes |
| 18. Paraguay | Yes | Yes | Yes | Yes | Yes | Yes | Yes |
| 19. Peru | Yes | Yes | Yes | Yes | Yes | No | Yes |
| 20. Suriname | Yes | No | Yes | Yes | No | No | No |
| **Total Yes** | **20** | **15** | **15** | **16** | **17** | **11** | **15** |
| **Total Countries per policy indicator** | **20** | **19** | **19** | **20** | **19** | **18** | **19** |
| **Total %age** | **0.100** | **0.789** | **0.789** | **0.800** | **0.894** | **0.611** | **0.789** |

| **WHO Region of the Americas** | | | | |
| --- | --- | --- | --- | --- |
| **Countries** | **Laws and regulations allow minor adolescents to seek** | | | **User fee waiver in public health sector for adolescents (15-19 years)** |
|  | **Contraceptives services except sterilization** | **Emergency contraception** | **Harm reduction intervention for injectable drug users** |  |
| 1. Argentina | Yes | Yes | No | Yes |
| 2. Belize | No | Not enquired | Unknown/not reported | Not enquired |
| 3. Bolivia (Plurinational State of) | Unknown/not reported | No | No | Yes |
| 4. Brazil | Yes | Yes | Yes | Yes |
| 5. Colombia | Yes | Yes | No | No |
| 6. Costa Rica | Yes | No | No | Yes |
| 7. Cuba | Yes | Yes | Unknown/not reported | Yes |
| 8. Ecuador | Yes | Yes | Yes | Yes |
| 9. El Salvador | Yes | Yes | Yes | Yes |
| 10. Guatemala | Yes | Yes | No | Yes |
| 11. Guyana | Yes | Not enquired | No | Not enquired |
| 12. Haiti | No | No | No | No |
| 13. Honduras | Yes | No | Unknown/not reported | No |
| 14. Jamaica | Yes | Yes | No | Yes |
| 15. Mexico | Yes | Yes | No | Yes |
| 16. Nicaragua | No | Not enquired | No | Not enquired |
| 17. Panama | Yes | Yes | Yes | No |
| 18. Paraguay | Yes | Yes | Yes | Yes |
| 19. Peru | No | Not enquired | No | Not enquired |
| 20. Suriname | Yes | Not enquired | No | Not enquired |
| **Total Yes** | **15** | **11** | **5** | **11** |
| **Total Countries per policy indicator** | **19** | **15** | **17** | **15** |
| **Total %age** | **0.789** | **0.733** | **0.294** | **0.733** |

Number of Countries included: 20

Number of Countries not included: 15 (Antigua and Barbuda, Bahamas, Barbados, Canada, Chile, Dominica, Dominican Republic, Grenada, St. Kitts and Nevis, St. Lucia, St. Vincent and the Grenadines, Trinidad and Tobago, USA, Uruguay, Venenzuela (Bolivarian Republic of)

Total number of Countries in the region: 35

| WHO Eastern Mediterranean Region | | | | | | | |
| --- | --- | --- | --- | --- | --- | --- | --- |
| Countries | National policies, strategies and plans for Adolescents | | | | | | |
|  | Sexual and reproductive health & family planning | Nutritional Intervention | Alcohol use prevention | Tobacco control activities | Mental health | Injury prevention | Violence |
| 1. Afghanistan | Yes | No | No | Yes | No | No | No |
| 2. Djibouti | No | No | No | No | No | No | No |
| 3. Egypt | No | Yes | No | Yes | Yes | Yes | Yes |
| 4. Iraq | Yes | Yes | Yes | Yes | Yes | Yes | Yes |
| 5. Morocco | Yes | Yes | Yes | Yes | Yes | Unknown/not reported | No |
| 6. Oman | Yes | Yes | Yes | Yes | Yes | Yes | Yes |
| 7. Pakistan | No | Yes | Yes | Yes | Yes | Yes | Yes |
| 8. Somalia | Yes | Yes | No | No | Yes | No | Unknown/not reported |
| 9. Sudan | Yes | Yes | No | Yes | Yes | No | No |
| 10. Yemen | Yes | Yes | No | No | No | No | No |
| **Total Yes** | **7** | **8** | **4** | **7** | **7** | **4** | **4** |
| **Total Countries per policy indicator** | **10** | **10** | **10** | **10** | **10** | **9** | **9** |
| **Total %age** | **0.700** | **0.800** | **0.400** | **0.700** | **0.700** | **0.444** | **0.444** |

| **WHO Eastern Mediterranean Region** | | | | |
| --- | --- | --- | --- | --- |
| **Countries** | **Laws and regulations allow minor adolescents to seek** | | | **User fee waiver in public health sector for adolescents (15-19 years)** |
|  | **Contraceptives services except sterilization** | **Emergency contraception** | **Harm reduction intervention for injectable drug users** |  |
| 1. Afghanistan | Unknown/not reported | Unknown/not reported | Unknown/not reported | Yes |
| 2. Djibouti | No | No | No | No |
| 3. Egypt | No | No | No | Yes |
| 4. Iraq | No | No | No | No |
| 5. Morocco | No | No | Unknown/not reported | No |
| 6. Oman | No | No | Unknown/not reported | No |
| 7. Pakistan | No | Unknown/not reported | Unknown/not reported | No |
| 8. Somalia | No | No | No | Unknown/not reported |
| 9. Sudan | No | No | No | No |
| 10. Yemen | No | No | No | No |
| **Total Yes** | **0** | **0** | **0** | **2** |
| **Total Countries per policy indicator** | **9** | **8** | **6** | **9** |
| **Total %age** | **0** | **0** | **0** | **0.222** |

Number of Countries included: 10

Number of Countries not included: 12 (Bahrain, Iran (Islamic Republic of), Jordan, Kuwait, Lebanon, Libya, Palestine, Qatar, Saudi Arabia, Syrian Arab Republic, Tunisia, United Arab Emirates)

Total number of Countries in the region: 22

| **WHO European Region** | | | | | | | |
| --- | --- | --- | --- | --- | --- | --- | --- |
| **Countries** | **National policies, strategies and plans for Adolescents** | | | | | | |
|  | **Sexual and reproductive health & family planning** | **Nutritional Intervention** | **Alcohol use prevention** | **Tobacco control activities** | **Mental health** | **Injury prevention** | **Violence** |
| 1. Albania | Yes | Yes | Yes | Yes | Yes | Yes | Yes |
| 2. Armenia | Yes | No | Yes | Yes | Yes | Yes | Yes |
| 3. Azerbaijan | Yes | Yes | Yes | Yes | Yes | Yes | Yes |
| 4. Bulgaria | Yes | Yes | No | Yes | Yes | Yes | Yes |
| 5. Kazakhstan | Yes | Yes | Yes | Yes | Yes | Yes | Yes |
| 6. Kyrgyzstan | Yes | Yes | Unknown/not reported | Yes | No | Yes | Yes |
| 7. Republic of Moldova | Yes | Yes | Yes | Yes | Yes | Yes | Yes |
| 8. Tajikistan | Yes | Yes | Yes | Yes | Yes | Yes | Yes |
| 9. Turkey | Yes | Yes | Yes | Yes | Yes | Yes | Yes |
| 10. Turkmenistan | Yes | No | No | No | No | No | Unknown/not reported |
| 11. Uzbekistan | Yes | Yes | Yes | Yes | Yes | Yes | Yes |
| **Total Yes** | **11** | **9** | **8** | **10** | **9** | **10** | **10** |
| **Total Countries per policy indicator** | **11** | **11** | **10** | **11** | **11** | **11** | **10** |
| **Total %age** | **0.100** | **0.818** | **0.800** | **0.909** | **0.818** | **0.909** | **0.100** |

| **WHO European Region** | | | | |
| --- | --- | --- | --- | --- |
| **Countries** | **Laws and regulations allow minor adolescents to seek** | | | **User fee waiver in public health sector for adolescents (15-19 years)** |
|  | **Contraceptives services except sterilization** | **Emergency contraception** | **Harm reduction intervention for injectable drug users** |  |
| 1. Albania | Unknown/not reported | Not enquired | Unknown/not reported | Not enquired |
| 2. Armenia | No | No | No | Yes |
| 3. Azerbaijan | No | No | No | Yes |
| 4. Bulgaria | Yes | Yes | No | No |
| 5. Kazakhstan | No | No | No | Yes |
| 6. Kyrgyzstan | Yes | Yes | Yes | No |
| 7. Republic of Moldova | Yes | Yes | Yes | No |
| 8. Tajikistan | Yes | Yes | No | No |
| 9. Turkey | Yes | Not enquired | Unknown/not reported | Not enquired |
| 10. Turkmenistan | No | Not enquired | No | Not enquired |
| 11. Uzbekistan | Yes | Yes | Unknown/not reported | Yes |
| **Total Yes** | **6** | **5** | **2** | **4** |
| **Total Countries** | **10** | **8** | **8** | **8** |
| **Total %age** | **0.600** | **0.625** | **0.250** | **0.500** |

Number of countries included: 11

Number of countries not included: 41 (Andorra, Austria, Belarus, Belgium, Bosnia and Herzegovina, Croatia, Cyprus, Czech Republic, Denmark, Estonia, Finland, France, Georgia, Germany, Greece, Hungary, Iceland, Ireland, Israel, Italy, Latvia, Lithuania, Luxembourg, Malta, Monaco, Montenegro, Netherlands, Norway, Poland, Portugal, Romania, Russian Federation, San Marino, Serbia, Slovakia, Slovenia, Spain, Sweden, Switzerland, The former Yugoslav Republic of Macedonia, Ukraine, United Kingdom)

Total number of countries in the region: 52

| **WHO South-East Asia Region** | | | | | | | |
| --- | --- | --- | --- | --- | --- | --- | --- |
| **Countries** | **National policies, strategies and plans for Adolescents** | | | | | | |
|  | **Sexual and reproductive health & family planning** | **Nutritional Intervention** | **Alcohol use prevention** | **Tobacco control activities** | **Mental health** | **Injury prevention** | **Violence** |
| 1. Bangladesh | Yes | Yes | Yes | Yes | Yes | Yes | Unknown/not reported |
| 2. India | Yes | Yes | Yes | Yes | Yes | Yes | Yes |
| 3. Indonesia | No | Yes | Yes | Yes | Yes | No | Yes |
| 4. Maldives | Yes | Yes | No | Yes | Yes | Yes | Yes |
| 5. Myanmar | Yes | Yes | Unknown/not reported | Unknown/not reported | Unknown/not reported | Unknown/not reported | Unknown/not reported |
| 6. Nepal | Yes | Yes | Yes | Yes | Yes | Yes | Yes |
| 7. Sri Lanka | Yes | Yes | Yes | Yes | Yes | No | No |
| 8. Timor Leste | Yes | Yes | Yes | Yes | Yes | Yes | Yes |
| **Total Yes** | **7** | **8** | **6** | **7** | **7** | **5** | **5** |
| **Total Countries per policy indicator** | **8** | **8** | **7** | **7** | **7** | **7** | **6** |
| **Total %age** | **0.875** | **0.100** | **0.857** | **0.100** | **0.100** | **0.714** | **0.833** |

| **WHO South-East Asia Region** | | | | |
| --- | --- | --- | --- | --- |
| **Countries** | **Laws and regulations allow minor adolescents to seek** | | | **User fee waiver in public health sector for adolescents (15-19 years)** |
|  | **Contraceptives services except sterilization** | **Emergency contraception** | **Harm reduction intervention for injectable drug users** |  |
| 1. Bangladesh | Unknown/not reported | Unknown/not reported | Unknown/not reported | No |
| 2. India | No | No | No | Yes |
| 3. Indonesia | No | No | Yes | No |
| 4. Maldives | No | Not enquired | Unknown/not reported | Not enquired |
| 5. Myanmar | Yes | Yes | Unknown/not reported | Yes |
| 6. Nepal | No | No | No | Yes |
| 7. Sri Lanka | Yes | Yes | No | Yes |
| 8. Timor Leste | No | No | No | Yes |
| **Total Yes** | **2** | **2** | **1** | **5** |
| **Total Countries per policy indicator** | **7** | **6** | **5** | **7** |
| **Total %age** | **0.286** | **0.333** | **0.200** | **0.714** |

Number of countries included: 8

Number of countries not included: 3 (Bhutan, Democratic Peoples Republic of Korea, Thailand)

Total number of countries in the region: 11

| **WHO Western Pacific Region** | | | | | | | |
| --- | --- | --- | --- | --- | --- | --- | --- |
| **Countries** | **National policies, strategies and plans for Adolescents** | | | | | | |
|  | **Sexual and reproductive health & family planning** | **Nutritional Intervention** | **Alcohol use prevention** | **Tobacco control activities** | **Mental health** | **Injury prevention** | **Violence** |
| 1. Cambodia | Yes | Yes | Yes | Yes | No | Yes | Yes |
| 2. China | Yes | Yes | Yes | Yes | Yes | Yes | Yes |
| 3. Kiribati | Yes | Yes | Yes | Yes | Yes | No | Yes |
| 4. Lao PDR | No | No | No | Yes | No | No | No |
| 5. Malaysia | Yes | Yes | Yes | Yes | Yes | Yes | Yes |
| 6. Papua New Guinea | Yes | No | Yes | Yes | No | No | Yes |
| 7. Philippines | Yes | Yes | No | No | No | Yes | Yes |
| 8. Solomon Islands | Yes | No | No | Yes | Unknown/not reported | No | No |
| 9. Vanuatu | Yes | No | Yes | Yes | Yes | No | No |
| 10. Viet Nam | Yes | Yes | No | Yes | No | Yes | Yes |
| **Total Yes** | **9** | **6** | **6** | **9** | **4** | **5** | **7** |
| **Total Countries per policy indicator** | **10** | **10** | **10** | **10** | **9** | **10** | **10** |
| **Total %age** | **0.900** | **0.600** | **0.600** | **0.900** | **0.444** | **0.500** | **0.700** |

| **WHO Western Pacific Region** | | | | |
| --- | --- | --- | --- | --- |
| **Countries** | **Laws and regulations allow minor adolescents to seek** | | | **User fee waiver in public health sector for adolescents (15-19 years)** |
|  | **Contraceptives services except sterilization** | **Emergency contraception** | **Harm reduction intervention for injectable drug users** |  |
| 1. Cambodia | Yes | Yes | Yes | No |
| 2. China | Unknown/not reported | Not enquired | Unknown/not reported | Not enquired |
| 3. Kiribati | Yes | Yes | Yes | Yes |
| 4. Lao PDR | No | No | No | Yes |
| 5. Malaysia | Yes | Yes | Yes | No |
| 6. Papua New Guinea | Yes | Yes | Yes | Yes |
| 7. Philippines | No | No | No | No |
| 8. Solomon Islands | No | No | No | Yes |
| 9. Vanuatu | Yes | Yes | No | No |
| 10. Viet Nam | Yes | Unknown/not reported | Yes | No |
| **Total Yes** | **6** | **5** | **5** | **4** |
| **Total Countries per policy indicator** | **9** | **8** | **9** | **9** |
| **Total %age** | **0.667** | **0.625** | **0.556** | **0.444** |

Number of countries included: 10

Number of countries not included: 17 (Australia, Brunei Darussalam, Cook Islands, Fiji, Japan, Marshall Islands, Micronesia (Federated States of), Mongolia, Nauru, New Zealand, Niue, Palau, Republic of Korea, Samoa, Singapore, Tonga, Tuvalu)

Total number of countries in the region: 27

**Appendix 7**: Evidence-based NCD health interventions for adolescents and lifestyle factors among adolescents to prevent adulthood NCDs

*Community-based interventions:* Community-based interventions view the health of people as embedded within the behaviours, norms and values of the community around them (1). In turn, they seek to instill community-level behavioural change by focusing on the diffusion of protective behaviours, social norms and influences.^27^ Examples of community-based interventions include educational programs, media campaigns, community mobilization, and positive youth development programs. These interventions have contributed to enhanced life skills, positive attitudes, social and emotional skills, and problem solving skills among adolescents (1). They have also had a positive impact on issues such as alcohol use, sexual behaviour, early pregnancy and early marriage (2–8). Community based interventions aimed at promoting gender-equitable norms have been the most commonly evaluated in LMICs. However, evidence on their effectiveness is mixed (9).

Other forms of community-based interventions seek to address conspicuous, emerging, and chronic health issues by providing preventative and curative health interventions through community platforms such as youth centres and adolescent-friendly clinics. Services delivered through youth centres include sexual and reproductive health counselling, access to modern methods of contraception, nutritional interventions for pregnant adolescents, and treatments for depression and anxiety (8). However, there is insufficient evidence on the impact of youth centres on adolescent health outcomes due to the limited access of adolescents to these services (8). Adolescent-friendly clinics seek to provide accessible, acceptable, equitable, appropriate and effective health services tailored to adolescents. Some evidence suggests their effectiveness in positively impacting condom use and the use of modern contraceptive methods among adolescents (4).

*School-based interventions:* School-based interventions use schools as a setting for targeting health risk factors among adolescents. Effective school-based interventions can be grouped into curriculum-based interventions and school-based health service interventions. Curriculum-based interventions have led to enhanced knowledge and attitudes around sexual and reproductive health and improved safe sex behaviour among adolescents in HICs and LMICs (5,10–15). They have also resulted in increased graduation and employment rates, and reduced heavy drinking, violence, and crime rates (16–20). Despite some evidence on the positive impact of curriculum-based interventions on suicide prevention, only a few studies have sought to determine their effectiveness (21–24). Overall, evidence on the impact of school-based interventions on adolescent mental health is lacking (5), and their implementation has been concentrated mainly in HICs (25).

School-based health service interventions entail delivering primary care interventions to students, such as contraceptives, vaccines, and micronutrient supplementation. The provision of contraceptives has generally shown mixed results in terms of its impact on safe sex behaviours among adolescents (12,26,27). By contrast, delivering vaccines such as human papillomavirus (HPV) vaccines in school settings has resulted in high coverage and acceptability among adolescents in LMICs (26,28,29). The distribution of iron and folic acid supplementation in schools has also had a positive impact in reducing anaemia prevalence among adolescent girls in LMICs (30).

*Peer-based interventions:* Peer-based interventions target risk factors stemming from peer relationships, and often take place in schools and community settings where such relationships are forged (1,31). These interventions generally consist of improving decision making, goal setting, anger management, and communication skills, as well as enhancing coping mechanisms in response to media and peer pressure. They further seek to improve interpersonal skills and encourage the forming of healthy relationships. Peer-based interventions have had a positive impact on substance use, delinquency, course failure, and school suspension among adolescents in HICs.^62-67^ As well, they have resulted in increased knowledge of sexual health and risky sexual behaviour among adolescents in LMICs (32–34).

*Family-based interventions:* Family-based interventions seek to enhance protective factors within family settings. They consist of improving the health and behaviours of first-time mothers during pregnancy, as well as strengthening parenting skills, reducing parental conflict, and promoting healthy relationships between parents and their children (31). In this sense, family-based interventions have the potential to address risk factors across developmental stages, from pre-birth to secondary school, as well as during parenthood (31). Outcomes include reduced child abuse and neglect, increased high school graduation rates, and decreased depression, alcohol use, drug use, risky sexual behaviour, adolescent pregnancies, and crime rates (2,35–48). Outcomes during parenthood include fewer births, reduced alcohol use, fewer sexual partners, lower welfare dependence and increased involvement in the workforce (44). Nonetheless, trials for family-based interventions have been conducted primarily in the United States (31), illustrating the need for further research on their impact in other countries, particularly in LMICs.

*Towards a multi-level, multi-component and intersectoral approach to improve NCD-related risk factors among adolescents:* The impact of the aforementioned interventions can be enhanced when implemented as part of a multi-level, multi-component and intersectoral approach (1). It is important to highlight the complexity in conceptualizing, implementing, and assessing such an approach, given the need for transdisciplinary research efforts, intersectoral collaborations, and significant time investments (49). Nonetheless, by capturing the complex pathways through which health risks and protective factors trickle down from the distal to the individual level and by fostering coordination across sectors, such multifaceted interventions are more likely to maximize impact and yield long-term improvements in NCD outcomes (49). Specific interventions as used in various settings and platforms and detailed in the table below.

| **Delivery platform** | **Intervention** | **Target population** | **Country implemented** | **Outcomes** |
| --- | --- | --- | --- | --- |
| **Community-based** | Seattle Social Development Project | Students attending schools in high crime neighbourhoods | United States | *Age 18:*  · Less violence  · Less heavy drinking  · Less likely to engage in intercourse  · Less likely to be pregnant or cause pregnancy  *Age 21:*  · Delayed onset of first intercourse  · Increase condom use  · Fewer sex partners  · Less likely to sell drugs  · More likely to graduate  *Age 27:*  · Better educational and economic attainment  · Less likely to be diagnosed with mental disorder  · Less likely to be diagnosed with an STI |
|  | Sistering, Informing, Healing, Loving and Empowering (SiHLE) | Sexually experienced girls aged 14-18 | United States | · Increased use of condoms,  · Decreased number of sexual partners,  · Fewer STIs (i.e., Chlamydia infections)  · Decrease in pregnancy |
|  | Project P.A.T.H.S. (Positive Adolescent Training through Holistic Social Programs) | Students in secondary school (Grades 7-9) | Hong Kong | · Better psychosocial competencies  · Reduced use of substances  · Reduced delinquency |
|  | Program H (Horizons) | Men and Women | Brazil  India, Tanzania, Croatia, Vietnam and several countries in Central America. | · Prevented intimate partner and sexual violence against women |
|  | Stepping Stones | Age 15-26 | South Africa | · Lower rates of intimate partner violence  · Reduced proportion of reported perpetration of intimate partner violence |
|  | Life skills Training | Youth from urban and suburban areas | United States | *After 3 years:*  · Reduction in drug use  *After 6 years:*  · Reduced one pack a-day smoking  · Reduced binge drinking  · Reduced illicit drug use (inhalants, narcotics, and hallucinogens |
| **School-based** | Children’s Aid Society Carrera | Boys and Girls aged 10-12 | United States | · Reduced teenage pregnancy |
|  | Unplugged | Students in grades 7-9 (aged 12-14) | 7 European Countries | · Reduction in drunkenness  · Reduction in the frequency of marijuana use |
|  | Signs of Suicide (SOS) | Adolescents aged 13 to 17 | United States, Canada and Ireland | · Decrease in suicide attempts |
|  | Good Behavior Game (GBG) | Late Childhood – ages 5 to 11 | United States  And Canadian provinces | · Decrease in suicide attempts |
|  | Olweus Bullying Prevention Program | Late Childhood – ages 5 to 11  Early Adolescence – ages 12 to 14  Late Adolescence - ages 15 to 18 | Norway  Australia, Lithuania,  Netherlands, United Kingdom and United States | · Decrease in being bullied (victims) and being perpetrators |
|  | Gatehouse project | Students in grade 8 | Australia | · Reduction in sexual activity, smoking, alcohol use and antisocial behaviour |
|  | Healthy Relationships | Adolescent males and females | Canada | · Decrease in perpetration and victimization of dating violence |
|  | Safe Dates | Adolescent males and females | Canada and United States | · Decrease in dating sexual violence |
|  | Shifting Boundaries | Adolescent males and females | United States | · Decrease in dating sexual violence |
| **Peer-based** | Peer provider reproductive health service | Youths <20 | United States | · Increased use of contraceptives  · Increased use of health care services  · Reduced incidence of pregnancy |
|  | Project ALERT | Middle and junior high school students from urban, suburban and rural areas | United States | · Reduced incidence of smoking  · Decreased use of alcohol and substance use |
| **Family-based** | Strengthening Families Program | Parents and Youth 10-14 | United States | *After a five-year follow-up:*  · Reduction in substance use and delinquency |
|  | New Beginnings Mother and Child Program | Youth 9-12 from divorces families | United States | *After a 6-year follow up:*  · Fewer sexual partners  · Fewer mental health symptoms  · Fewer externalizing problems  · Fewer substance-misuse disorders |
|  | The Nurse-Family Partnership | Low-income first time mothers | United States | *Mothers:*  · Fewer pregnancies  · Fewer self-reported arrests  · Less smoking during pregnancy  *At age 15:*  · Fewer arrests and convictions  · Fewer drinking days  · Fewer lifetime sexual partners  *Children:*  · Improved cognitive development  · Fewer serious behaviour problems |
|  | Functional Family Therapy | Youth and families | United States | · Reductions in substance use and delinquency |
|  | Positive Parenting Program (Triple P) | Infants ages 0 to 2    Early Childhood – ages 3 to 4    Late Childhood ages 5 to 11) | United States and Australia | · Reduce child maltreatment |
| **Multi-level, Multi-component and intersectoraal approach** | Zomba Cash Transfer Program | Females aged 13-23 | Malawi | *At a 1-year follow up:*  · Recent dropouts more likely to return to school and stay in school  *Overall:*  · Decrease in early marriage |
|  | Conditional Cash Transfer | Students in grade 6 (age 14) | Kenya | *At a 3-year follow up:*  · Less likely to have ever had sexual intercourse (girls)  · Less likely to have dropped out of school (girls)  · Less likely be married (boys) |
|  | Saving bonds program | Girls younger than 18 years of age | India | · Increased educational attainment |
|  | Computer-Based Intervention | Youth aged 10-12 | United States | · Reduction in smoking, drinking and marijuana use, improving family involvement  · Reducing negative peer influences |
|  | KiVa | Students in grades 1 to 9 (Ages 7 to 15) | Finland | · Reduced physical victimization and cybervictimization |

**Appendix 8:** References

1. Patton GC, Sawyer SM, Santelli JS, Ross DA, Afi R, Allen NB, et al. Our future : a Lancet commission on adolescent health and wellbeing. 2016;6736(16).

2. Carson, K.V., Brinn, M.P., Labiszewski, N.A., Esterman, A.J., Chang, A.B., & Smith BJ. Community interventions for preventing smoking in young people. Cochrane Database Syst Rev. 2000;(7):2–4.

3. Foxcroft, D. R., & Tsertsvadze A. Universal family‐based prevention programs for alcohol misuse in young people. Cochrane Libr. 2011;

4. Gottschalk LB, Ortayli N. Interventions to improve adolescents’ contraceptive behaviors in low- and middle-income countries: A review of the evidence base. Contraception. The Authors; 2014;90(3):211–25.

5. Lassi ZS, Salam RA, Das JK, Wazny K, Bhutta ZA. An unfinished agenda on adolescent health: Opportunities for interventions. Semin Perinatol. 2015;39(5):353–60.

6. Martineau F, Tyner E, Lorenc T, Petticrew M, Lock K. Population-level interventions to reduce alcohol-related harm: An overview of systematic reviews. Prev Med (Baltim). Elsevier Inc.; 2013;57(4):278–96.

7. Thomas RE, Lorenzetti DL, Spragins W. Systematic review of mentoring to prevent or reduce tobacco use by adolescents. Acad Pediatr. Elsevier Ltd; 2013;13(4):300–7.

8. Zuurmond MA; Geary RS; Ross DA. The effectiveness of youth centers in increasing use of sexual and reproductive health services: a systematic review. Stud Fam Plann. 2012;43(4):239–54.

9. Lundgren R, Amin A. Addressing intimate partner violence and sexual violence among adolescents: Emerging evidence of effectiveness. J Adolesc Heal. Elsevier Inc.; 2015;56(1):S42–50.

10. Allen-Meares P, Montgomery KL, Kim JS. School-based social work interventions: A cross-national systematic review. Soc Work (United States). 2013;58(3):253–62.

11. Amaugo LG, Papadopoulos C, Ochieng BMN, Ali N. The effectiveness of HIV/AIDS school-based sexual health education programmes in Nigeria: A systematic review. Health Educ Res. 2014;29(4):633–48.

12. Blank L, Baxter SK, Payne N, Guillaume LR, Squires H. Systematic review and narrative synthesis of the effectiveness of contraceptive service interventions for young people, delivered in health care settings. Health Educ Res. 2012;27(6):1102–19.

13. Fonner VA, Armstrong KS, Kennedy CE, O’Reilly KR, Sweat MD. School based sex education and HIV prevention in lowand middle-income countries: A systematic review and meta-analysis. PLoS One. 2014;9(3).

14. Harrison A, Newell M-L, Imrie J, Hoddinott G. HIV prevention for South African youth: which interventions work? A systematic review of current evidence. BMC Public Health. 2010;10(1):102.

15. A MKSRG. The efficacy of interventions to reduce adolescent childbearing in low- and middle-income countries: a systematic review. TT -. Stud Fam Plann. 2013;44(4):369–88.

16. Hawkins JD, Catalano RF, Kosterman R, Abbott R, Hill KG. Preventing adolescent health-risk behaviors by strengthening protection during childhood. Arch Pediatr Adolesc Med. 1999;153(3):226–34.

17. Hawkins JD, Guo J, Hill KG, Battin-Pearson S, Abbott RD. Long-term effects of the Seattle social development intervention on school bonding trajectories. Appl Dev Sci. 2001;5(4):214–24.

18. Hawkins JD, Kosterman R, Catalano RF, Hill KG, Abbott RD. Promoting Positive Adult Functioning Through Social Development Intervention in Childhood. Arch Pediatr Adolesc Med. 2005;159(1):25–31.

19. Hawkins, J. D., Kosterman, R., Catalano, R. F., Hill, K. G., & Abbott RD. Effects of social development intervention in childhood 15 years later. Arch Pediatr Adolesc Med. 2008;162(12):1133–41.

20. Lonczak HS, Abbott RD, Hawkins JD, Kosterman R, Catalano RF. Effects of the Seattle Social Development Project on Sexual Behavior, Pregnancy, Birth, and Sexually Transmitted Disease Outcomes by Age 21 Years. Arch Pediatr Adolesc Med. 2002;156(5):438.

21. Cusimano MD, Sameem M. The effectiveness of middle and high school-based suicide prevention programmes for adolescents: A systematic review. Inj Prev. 2011;17(1):43–9.

22. Katz C, Bolton SL, Katz LY, Isaak C, Tilston-Jones T, Sareen J. A systematic review of school-based suicide prevention programs. Depress Anxiety. 2013;30(10):1030–45.

23. Klimes-Dougan B, Klingbeil DA, Meller SJ. The impact of universal suicide-prevention programs on the help-seeking attitudes and behaviors of youths. Crisis. 2013;34(2):82–97.

24. Robinson J, Cox G, Malone A, Williamson M, Baldwin G, Fletcher K, et al. A systematic review of school-based interventions aimed at preventing, treating, and responding to suicide- related behavior in young people. Crisis. 2013;34(3):164–82.

25. World Health Organization. Adolescent mental health: mapping actions of nongovernmental organizations and other international development organizations. Geneva; 2012.

26. Binagwaho, A., Wagner, C.M., Gatera, M., Karema, C., Nutt, C.T., Ngabo F. Achieving high coverage in Rwanda’s national human papillomavirus vaccination programme. Bull World Health Organ. 2012;623–8.

27. Owen J, Carroll C, Cooke J, Formby E, Hayter M, Hirst J, et al. School-linked sexual health services for young people (SSHYP): A survey and systematic review concerning current models, effectiveness, cost-effectiveness and research opportunities. Health Technol Assess (Rockv). 2010;14(30).

28. LaMontagne, D.S., Barge, S., Thi Le, N., Mugisha, E., Penny, M.E., Gandhi, S., Janmohamed, A., Kumakech, E., Mosquiera, N.R., Nguyen, N.Q., Paul, P., Tang, Y., Minh, T.H., Uttekar, B.P., & Jumaan AO. Human papillomavirus vaccine delivery strategies that achieved high coverage in low- and middle-income countries. Bull World Health Organ. 2011;89(11):821–30.

29. Joel Ladner, Marie Helene Besson, Marian Rodrigues, Etienne Audureau, Joseph Saba. Performance of 21 HPV vaccination programs implemented in low and middle-income countries , 2009 – 2013. BMC Public Health. 2014;14:1–11.

30. HAIDER R. Adolescent nutrition: a review of the situation in selected South‐East Asian countries. New Delhi; 2006.

31. Catalano RF, Fagan AA, Gavin LE, Greenberg MT, Irwin CE, Ross DA, et al. Worldwide application of prevention science in adolescent health. Lancet. Elsevier Ltd; 2012;379(9826):1653–64.

32. Speizer IS, Magnani RJ, Colvin CE. The effectiveness of adolescent reproductive health interventions in developing countries: A review of the evidence. J Adolesc Heal. 2003;33(5):324–48.

33. Fenton KA, Johnson AM, Mcmanus S, Erens B. Series editors Measuring sexual behaviour : methodological challenges in survey research. Sex transm Inf. 2001;84–92.

34. Kirby, D., Obasi, A., & Laris BA. The effectiveness of sex education and HIV education interventions in schools in developing countries. World Heaolth Organ Tech Rep Ser. 2006;938(103):317–41.

35. Campbell FA, Ramey CT, Miller-johnson S. Early Childhood Education : Young Adult Outcomes From the Abecedarian Project Elizabeth Pungello and Joseph Sparling. Appl Dev Sci. 2002;6(1):42–57.

36. Schinke, S.P., Schwinn, T.M., Noia, J., & Cole KC. Reducing the risks of alcohol use among urban youth: three-year effects of a computer-based intervention with and without parent involvement. J Stud Alcohol. 2004;65(4):443–9.

37. Schweinhart, L. J., Barnes, H. V., & Weikhart DP. Significant benefits: The High/Scope Perry preschool study through age 27. In: Child Welfare: Major Themes in Health and Social Welfare. London; New York: Routledge; 2005. p. 9–29.

38. Schwinn, T. M., & Schinke SP. Preventing Alcohol Use Among Late Adolescent Urban Youth: 6-Year Results From a Computer-Based Intervention. J Stud Alcohol Drugs. 2010;71(4):535–8.

39. Thomas RE, Baker PRA, Thomas BC, Lorenzetti DL. Family-based programmes for preventing smoking by children and adolescents. Cochrane Database Syst Rev. 2015;2017(12).

40. Spoth R, Trudeau L, Guyll M, Shin C, Redmond C. Universal Intervention Effects on Substance Use Among Young Adults Mediated by Delayed Adolescent Substance Initiation. J Consult Clin Psychol. 2009;77(4):620–32.

41. Fagan AA, Catalano RF. What Works in Youth Violence Prevention: A Review of the Literature. Res Soc Work Pract. 2013;23(2):141–56.

42. Gates S, Mccambridge J, Smith LA, Foxcroft D. Interventions for prevention of drug use by young people delivered in non-school settings. Cochrane Database Syst Rev. 2009;(1).

43. Kumpfer KL, Alvarado R, Whiteside HO. Family-based interventions for substance use and misuse prevention. Subst Use Misuse. 2003;38(11–13):1759–1787+1916.

44. Controlled R, Olds D, Henderson CR, Cole R, Eckenrode J, Kitzman H, et al. Long-term Effects of Nurse Home Visitation on Children ’ s Criminal and Antisocial Behavior. 2014;280(14).

45. Olds, D.L., Kitzman, H., Cole, R., Robinson, J., Sidora, K., Luckey, D.W., Henderson, C.R., Hanks, C., Bondy, J. & Holmberg J. Effects of nurse home-visiting on maternal life course and child development: Age 6 follow-up results of a randomized trial. Pediatrics. 2004;114(6):1550–9.

46. Olds, D.L., Robinson, J., O’Brien, R., Luckey, D.W., Pettitt, L.M., Henderson, C.R., Ng, R.K., Sheff, K.L., Korfmacher, J., Hiatt, S. & Talmi A. Home visiting by paraprofessionals and by nurses: a randomized, controlled trial. Pediatrics. 2002;110(3):486–96.

47. Reynolds AJ, Temple JA, Ou S-R, Robertson DL, Mersky JP, Topitzes JW, et al. Effects of a School-Based, Early Childhood Intervention on Adult Health and Well-being. Arch Pediatr Adolesc Med. 2007;161(8):730.

48. Reynolds AJ, Temple J a, Robertson DL, Mann E a. Long-term Effects of an Early Childhood Intervention on Educational Achievement. 2001;285(18):2339–47.

49. Trickett EJ, Beehler S. The Ecology of Multilevel Interventions to Reduce Social Inequalities in Health. Am Behav Sci. 2013;57(8):1227–46.
